# Revealing Local Diffusion Dynamics in Hybrid Solid Electrolytes

**DOI:** 10.1021/acsenergylett.5c00214

**Published:** 2025-03-17

**Authors:** Shengnan Zhang, Leon Felix Mueller, Laurence Macray, Marnix Wagemaker, Lars J. Bannenberg, Swapna Ganapathy

**Affiliations:** † Section Storage of Electrochemical Energy, Radiation Science and Technology, Faculty of Applied Sciences, Delft University of Technology, Mekelweg 15, 2629 JB, Delft, The Netherlands

## Abstract

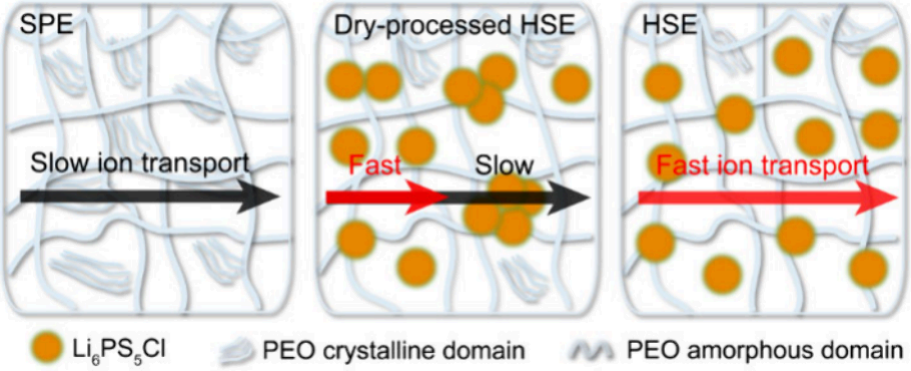

Hybrid solid electrolytes (HSEs) leverage the benefits of their organic and inorganic components, yet optimizing ion transport and component compatibility requires a deeper understanding of their intricate ion transport mechanisms. Here, macroscopic charge transport is correlated with local lithium (Li)-ion diffusivity in HSEs, using poly(ethylene oxide) (PEO) as matrix and Li_6_PS_5_Cl as filler. Solvent- and dry-processing methods were evaluated for their morphological impact on Li-ion transport. Through multiscale solid-state nuclear magnetic resonance analysis, we reveal that the filler enhances local Li-ion diffusivity within the slow polymer segmental dynamics. Phase transitions indicate inhibited crystallization in HSEs, with reduced Li-ion diffusion barriers attributed to enhanced segmental motion and conductive polymer conformations. Relaxometry measurements identify a mobile component unique to the hybrid system at low temperatures, indicating Li-ion transport along polymer–filler interfaces. Comparative analysis shows solvent-processed HSEs exhibit better morphological uniformity and enhanced compatibility with Li-metal anodes via an inorganic-rich solid electrolyte interphase.

Driven by the demand for high-energy-density and safe energy storage, research is increasingly pivoting from conventional liquid lithium (Li)-ion batteries toward all-solid-state Li-metal batteries.^1,2^ To achieve this, developing solid-state electrolytes with key attributes such as high ionic conductivity, mechanical stability, flexibility, and a wide operating temperature range is crucial.^3,4^ Among many solid-state electrolytes, poly(ethylene oxide) (PEO)-based systems show superior mechanical flexibility, processability, and electrode wettability. However, the low ionic conductivity at room temperature and insufficient mechanical strength pose challenges for practical use.^5−8^ As a combinatorial strategy, hybrid solid electrolytes (HSEs) that incorporate ceramic fillers into the PEO–Li salt (LiX) matrix have proven to be effective in improving ionic conductivity and mechanical properties. The added fillers can be either passive or active. While passive fillers function similarly to molecular plasticizers by reducing the crystallinity of the polymer, active fillers have an additional role that contributes to higher ionic conductivity by forming extra Li-ion transport pathways. These pathways can be established through the filler phase and the polymer–filler interface.^9^ Previous research has confirmed enhanced ion transport in the PEO-based HSEs when using active fillers such as garnet-type oxides,^10−12^ NASICON-type phosphates,^10^ and sulfides.^13−15^ Most research focuses on macroscopic and interfacial Li-ion conduction to access the contributions of each phase to ion transport,^14,16−18^ yet an in-depth understanding of local Li-ion dynamics, which are closely correlated to overall conductivity, is still lacking. Local Li-ion dynamics in HSEs pertain to the behavior of Li-ions at a microscopic level, which provides essential insight into the mechanisms governing ion movement and polymer–filler interactions within the HSEs.

To date, the room temperature conductivity of PEO-based HSEs remains unsatisfactory despite the use of active fillers expected to enhance overall conductivity through their intrinsic high ionic conductivity. This is typically attributed to the poor ionic conductivity of the PEO matrix and the limited participation of inorganic particles. The later is likely due to high energy barriers for interfacial Li-ion transport, which arise from space-charge effects at the polymer–ceramic interface and the presence of a rigidified polymer layer adhering to the particle surfaces, similar to those reported in PEO-garnet composite systems.^19,20^ While increasing the filler concentration can improve ionic conductivity, the resulting filler agglomeration often impedes Li-ion transport and reduces overall conductivity.^21^ Especially in systems with a low inorganic fraction, inadequate conductivity is often ascribed to limited Li-ion transport at the polymer–filler interface.^13,18,22,23^ However, the interface is intrinsically coupled with the polymer matrix, where ionic conduction within the matrix is integral to determining the overall conductivity. This aspect has received little attention, although it can greatly affect the Li-ion diffusion mechanism and dominate the overall conductivity. Thus, advancing HSEs requires understanding their local structure and ion dynamics within the polymer matrix. In this regard, the formulation method plays a pivotal role, as it can result in a large disparity in performance. Many reports describe the use of solvent-assisted preparation for HSEs, which generally outperforms dry-mixing in terms of homogeneity and ease of processing. However, the disadvantage is that solvent processing typically induces side reactions between the electrolytes and the solvent.^24,25^ Building on this, solid-state nuclear magnetic resonance (NMR) stands out as an effective technique for probing local chemical environments and dynamic behavior in HSEs across various time and length scales, with isotope selectivity and nondestructive analysis.^26,27^

Herein, we aim to understand local Li-ion mobility across different dynamic ranges in the HSE system built on a PEO-lithium-bis(trifluoromethanesulfonyl)imide (LiTFSI) matrix and the fast ion-conducting Li_6_PS_5_Cl filler, with a particular focus on the polymer phase. The impact of the Li_6_PS_5_Cl filler on local Li-ion dynamics in the PEO phase is compared to that of the pure PEO electrolyte (nonfilled), alongside an evaluation of solvent- and dry-processing methods in HSEs formulation. Temperature-dependent solid-state NMR line-width and relaxometry measurements, combined with electrochemical impedance spectroscopy (EIS), enable the quantification of local Li-ion mobility and its correlation with macroscopic charge transport. Results show that incorporating Li_6_PS_5_Cl filler improves Li-ion diffusion at both bulk and local length scales, which is attributed to inhibited crystallization and the alteration of the polymer chain conformation toward a more Li-ion conductive structure. When comparing formulation methods, dry-processed electrolytes display slower local Li-ion dynamics and inhomogeneous phase distribution, due to the morphological heterogeneity introduced by dry-mixing. Finally, adding Li_6_PS_5_Cl fillers has proven to enhance the compatibility of the PEO-based polymer electrolytes with the Li-metal anode and form a more conductive solid electrolyte interphase.

## Macroscopic Li-Ion Conduction

**Figure 1:**
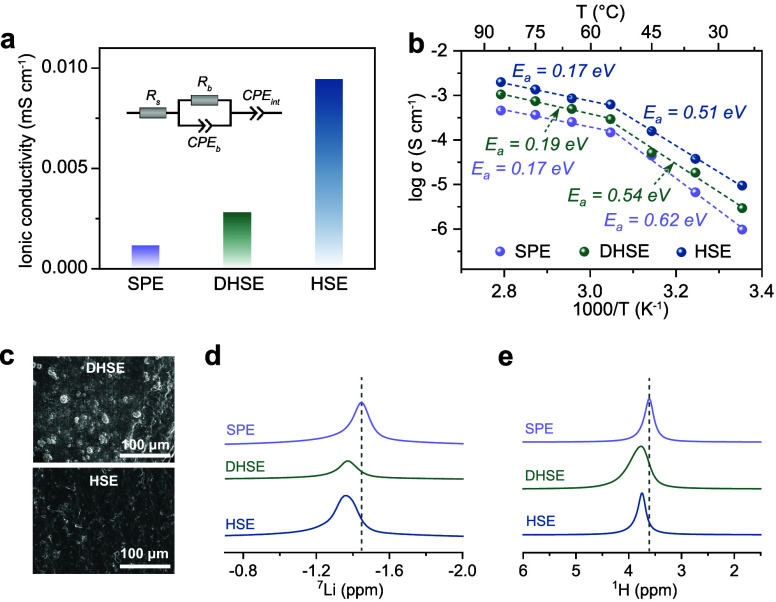
Characterization of the SPE and (D)HSE. (a) Ionic conductivity obtained by EIS of the cells using the SPE, DHSE, and HSE at 25 °C. (b) Arrhenius plots of the SPE, DHSE, and HSE with fitted activation energies (*E*_a_) corresponding to different processes. In the high-temperature region, the *E*_a_ values are 0.17 ± 0.02, 0.19 ± 0.01, and 0.17 ± 0.01 eV for the SPE, DHSE, and HSE, respectively. In the low-temperature region, the *E*_a_ values are 0.62 ± 0.02, 0.54 ± 0.03, and 0.51 ± 0.01 eV for the SPE, DHSE, and HSE, respectively. (c) SEM images of the DHSE and HSE. One-pulse (d) ^7^Li and (e) ^1^H magic angle spinning (MAS) solid-state NMR spectra of the SPE, DHSE, and HSE electrolytes. The ^7^Li spectra correspond to the LiTFSI-PEO resonance, with chemical shifts at −1.45, −1.38, and −1.37 ppm for the SPE, DHSE, and HSE, respectively. ^7^Li spectra over a broader range, including the Li_6_PS_5_Cl peak, are shown in Figure S3a. The ^1^H peak positions for the SPE, DHSE, and HSE are 3.61, 3.77, and 3.75 ppm, respectively.

The HSEs composed of PEO-LiTFSI (EO:Li = 18:1) and 10 wt % Li_6_PS_5_Cl (8 vol %) were fabricated using both solvent- and dry-processing methods, referred to as the HSE and DHSE, respectively (see details in Methods and Materials). The Li_6_PS_5_Cl particle size is ∼1.5 μm (see the scanning electron microscopy (SEM) image and size distribution analysis in Figure S1). To investigate the impact of Li_6_PS_5_Cl fillers on the PEO-LiTFSI solid polymer electrolyte (SPE) and evaluate the feasibility of the dry-processing approach for fabricating HSEs, their ionic conductivities at variable temperatures were measured using EIS. Evidently, the addition of the Li_6_PS_5_Cl filler notably improved the ionic conductivity of the SPE, with the solvent-processed HSE showing the highest ionic conductivity (Figure 1a). In the Arrhenius plot, two linear fits were applied due to a noticeable deflection after the melting temperature (*T*_m_) of the polymer matrix (Figure 1b). This deflection can be attributed to either the melting of the crystalline phase or the rearrangement of the −EO– moieties.^28^ The obtained activation energies (*E*_a_) reveal more distinct differences in the lower temperature region, with values of 0.62 (±0.02), 0.54 (±0.03), and 0.51 (±0.01) eV for the SPE, DHSE, and HSE, respectively. Since both cations and anions contribute to the measured conductivity, these values represent collective ion motion rather than solely Li-ion mobility. However, given that anions generally exhibit higher mobility, the *E*_a_ is predominantly influenced by Li-ion transport.^29,30^ This indicates that incorporating the Li_6_PS_5_Cl filler lowers the energy barrier for Li-ion diffusion in the SPE, particularly when the polymer matrix is more rigid. The consistently higher ionic conductivities of the (D)HSE compared to the SPE suggest reduced crystallinity in the (D)HSE. However, above 55 °C, the SPE likely becomes amorphous as well (*T*_m_ = 57.2 °C; see differential scanning calorimetry (DSC) results in Figure S2), indicating that additional mechanisms can be involved. Similar to the PEO–garnet systems, space-charge effects and noncovalent anion trapping at polymer–filler interfaces are potential contributors.^31,32^ The high ionic conductivity of Li_6_PS_5_Cl can create local Li-ion imbalance, forming space-charge regions, while its Li-ion-rich surface can immobilize TFSI^–^ anions through electrostatic interactions, thereby enhancing Li-ion transport. Furthermore, the solvent-processed HSE exhibits a lower *E*_a_ compared to its dry-processed counterpart, likely due to better homogeneity in the HSE, as reflected in the SEM images in Figure 1c, where the DHSE displays notable particle agglomeration on the surface compared with the smooth HSE.

Expanding on the aforementioned observations, one-pulse ^7^Li and ^1^H solid-state NMR were performed on the pristine membrane electrolytes, with the aim of elucidating the interactions between Li_6_PS_5_Cl and the polymer matrix and assessing the impact of processing methods. A downfield shift of the LiTFSI-PEO peak is detected in the ^7^Li spectra for both the HSE and DHSE (Figure 1d). The deshielded Li environment indicates reduced electron density around the Li atoms, resulting from decreased coordination with the −EO– units in the polymer backbone.^33,34^ Correspondingly, the ^1^H spectra show a downfield shift of the ^1^H peak in samples containing Li_6_PS_5_Cl, signifying a more deshielded ^1^H environment due to the less tightly bonded EO–Li coordination (Figure 1e). Although the chemical shifts were similar across different processing methods, the DHSE displays a weaker LiTFSI-PEO peak and a stronger Li_6_PS_5_Cl peak, indicating sample heterogeneity and possible filler agglomeration (Figure S3a). In addition, the broader ^1^H peak in the DHSE spectra suggests poorer uniformity within the sample.

## Local Li-Ion Dynamics

**Figure 2:**
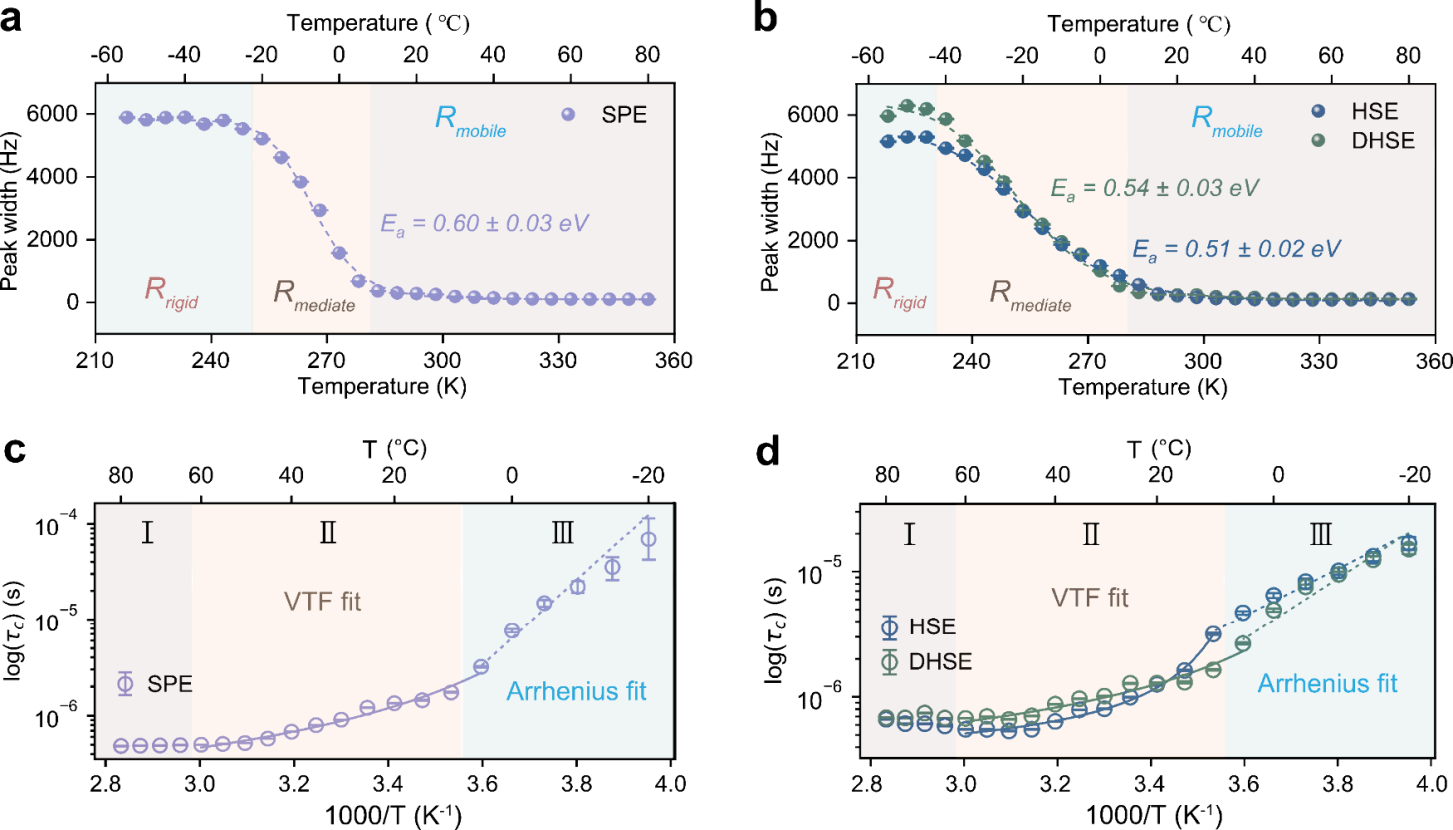
Probing local Li-ion dynamics using ^7^Li NMR line-width analysis. Temperature-dependent ^7^Li line widths fitted with the Hendrickson–Bray model^35^ (dashed line) for (a) the SPE and (b) the(D)HSE. Semilogarithmic plot of the correlation time (τ_c_) against the reciprocal temperature for (c) the SPE and (d) the (D)HSE, fitted with the Abragam model (see details in Supplementary Text 2).^36^ The different dynamical regimes (I–III) of the ^7^Li correlation times are marked by distinct fill colors: regime I corresponds to the polymer melt regime with fast Li-ion motion and correspondingly short τ_c_, regimes II and III are mixed-phase regimes fitted by the VTF and Arrhenius laws,^37^ respectively.

Having established the impact of filler addition and processing methods on the bulk ionic conductivity and the polymer–filler interactions, it is crucial to correlate the macroscopic ion transport and structures with the underlying dynamic changes at the molecular level for a fundamental understanding of the ion conduction mechanisms. To this end, NMR line-width measurements were employed to analyze the local structure and ion dynamics within the electrolytes, providing insights into the mechanisms behind the improved bulk conductivity and the influence of the processing method on local Li-ion diffusion in the HSEs. In the line-width measurements, increased mobility averages out dipolar interactions, resulting in narrower lines.^38^Figure S4 shows the deconvolution of the overlapping LiTFSI-PEO and Li_6_PS_5_Cl peaks, enabling more accurate determination of both line widths and amplitudes. The PEO phase is the primary focus of this study, as it serves as the matrix and has a great impact on the overall ionic conductivity of the HSEs. Figure 2a,b shows the change in line width as a function of temperature, distinguishing three behavioral regimes for both the SPE and (D)HSE: (i) Rigid lattice regime (*R*_rigid_): In this regime, the line widths are very broad (≥5000 Hz) and remain constant as the temperature decreases. This indicates low Li mobility, where the hopping frequency is slower than the rate of magnetic environment fluctuations responsible for line-broadening, which occurs when the system is below the glass transition temperature (*T*_g_). The broad peaks observed are a result of quadrupolar and dipole–dipole interactions.^39^ (ii) Intermediate regime (*R*_mediate_): This regime signals the onset of motional narrowing, characterized by a gradual decrease in line width. As the temperature increases, the Li-ion motion increases to a rate where the ion motion becomes comparable to the time scale of the NMR experiment (related to the Larmor frequency). Consequently, the local magnetic fields experienced by the nuclei start to average out. (iii) Fully narrowed regime (*R*_mobile_): In this regime, the line width becomes very narrow (≤100 Hz). Here, the Li-ions exhibit high mobility, leading to a complete averaging of the local magnetic fields. The rapid Li-ion motion causes the NMR measurements to detect only an averaged and diminished magnetic interaction. This indicates a high level of segmental motion and dynamic behavior within the PEO matrix. Comparing the SPE and (D)HSE reveals differences in the temperature ranges of their respective regimes. In the SPE, the onset of *R*_rigid_ occurs at a temperature of −20 °C, whereas in the (D)HSE, the onset of the rigid lattice regime occurs at temperatures as low as −45 °C. This corresponds to a wider temperature range (*ΔT*) for the *R*_mediate_ in the (D)HSE (*ΔT*_(D)HSE_ = 40 °C), compared to the SPE where narrowing occurs faster (*ΔT*_SPE_ = 25 °C). However, the fully narrowed regime *R*_mobile_ begins at approximately 5 °C for both samples. In the SPE, the shorter intermediate regime indicates a rapid decrease in Li-ion motion within the amorphous polymer phase below 5 °C, due to the immobilization induced by the growing crystalline phase. In contrast, the (D)HSE exhibits inhibited growth of the crystalline phase, allowing Li-ion motion to persist across a broader temperature range. This is attributed to the plasticizing effect and the inhibition of crystallization with the presence of fillers.^40,41^ In addition, the two processing methods also display a visible difference, notably with the dry method showing broader peaks compared with the solvent method at temperatures below −20 °C (Figure 2b). The broader peak suggests additional heteronuclear dipolar line-broadening arising from closer interactions between Li^+^ and TFSI^–^. This hints that the inherent morphology of the DHSE is characterized by incomplete Li salt solvation within the PEO domains due to inhomogeneous particle distribution, which in turn influences the charge distribution near the Li nuclei.

Taking a closer look at the line widths in the *R*_mobile_ regime (Figure S5), a second, smaller narrowing step is present for both the SPE and (D)HSE, despite the small line-width values. This decrease can be attributed to a gradual increase in the amorphous phase in the PEO matrix before *T*_m_, which is consistent with the *T*_m_ obtained from the DSC measurements (Figure S2). After *T*_m_ was reached, the line width remains constant for all samples. It is noteworthy that before reaching *T*_m_, the HSE exhibits the smallest line width of all of the samples, indicating accelerated Li dynamics as well as a more homogeneous chemical environment for Li-ions within the PEO matrix of the HSE at typical operational temperatures. Notably, the line width of the dry-processed sample remains larger also after melting, suggesting greater heterogeneity in chemical environments due to the dry-processing procedure. This indicates that the Li mobility in this temperature range is predominantly influenced by polymer segmental motion, with well-mixed Li_6_PS_5_Cl fillers enhancing the local chain mobility. Fitting the temperature-dependent line width using Hendrickson–Bray’s phenomenological equation (see details in Supplementary Text 1 and fitting parameters in Table S3),^35^ the activation energy (*E*_a_) of the motional process responsible for the line-narrowing can be determined. The *E*_a_ obtained from the NMR line-width measurement corresponds to local ion dynamics on the time scale of several microseconds.^38^ Both the HSE and DHSE show a lower *E*_a_ and a different curve shape compared to the SPE, with the DHSE exhibiting slightly higher *E*_a_ than the HSE. This suggests that the addition of a Li_6_PS_5_Cl filler enhances the Li-ion diffusion and lowers the energy barrier for Li-ion transport. It is worth noting that the line width reflects a convolution of interactions contributing to line broadening from both the crystalline, semicrystalline, and amorphous polymer phases, indicating that the Hendrickson–Bray’s model captures the averaged ion dynamics.

These local Li-ion dynamics were further analyzed with the Abragam model (see details in Supplementary Text 2), to determine the line-width proportional correlation time (τ_c_).^36,42^ This reveals how quickly ions can reorient themselves or move between different sites. In the temperature range analyzed, all of the electrolytes exhibit three distinct regimes of τ_c_, as indicated in Figure 2c,d. Within regime I, the polymer phase exists in a melted, fully amorphous state, where τ_c_ is short, and segmental dynamics dominate. Thermal motion of both the polymer backbone and Li leads to high-frequency changes in the EO-Li coordination shell. In the intermediate temperature regime (II), a noticeable curved trend emerges as the temperature decreases, indicating that Li-ion reorientation follows a Vogel–Tammann–Fulcher (VTF) process.^37^ This VTF curvature reflects increasing heterogeneity in the sample as it cools.^43^ The as-formed regions thus show distinct mobility characteristics: high-mobility amorphous fractions and low-mobility crystalline fractions. Cooperative rearrangement of larger polymer chain regions is crucial for Li-ion motion. In this semicrystalline state, the motion follows a superexponential behavior rather than an Arrhenius-type exponential relationship, attributed to increased cooperativity and heterogeneity within the system.^44^ While the polymer crystallizes, Li-ions do not remain within the crystalline fraction. Instead, a phase segregation occurs, as noted by Marzantowicz et al.,^45^ where the dissolved Li salt does not integrate well into the crystalline PEO structure and accumulates in the amorphous phase. A concentrated salt front develops at the boundary of the crystal phase as well as at interfaces with the filler particles, leading to an increased level of formation of PEO:LiTFSI complexes in the amorphous phase. This process creates domains of PEO:LiTFSI complexes within a semicrystalline electrolyte. Despite this phase formation, the semicrystalline electrolyte only marginally reduces mobility, as ions are infrequently trapped within the crystalline phase.^45^ Regime III is marked by a rapid change in line width. The τ_c_ of the Li nuclei now exhibits an Arrhenius temperature dependency, suggesting the presence of another phase transition. The steepness of the Arrhenius plot indicates a high energy barrier for altering the Li coordination shell. In this regime, the increase in τ_c_ is influenced not only by the crystalline fraction of the PEO phase but also by the crystallization of the PEO:LiTFSI complexes. Consequently, these crystalline phases contribute to a solid-like mechanism of ion transport.^46^

From the EIS measurements and the Hendrickson–Bray fits of the line width, the addition of Li_6_PS_5_Cl fillers demonstrates a reduced *E*_a_ for Li-ion diffusion. This reduction is also evident from the *E*_a_ values obtained from both regime II and regime III (see Table S1) obtained using the VTF and Arrhenius fits. It is worth noting that the *E*_a_ obtained from the VTF fit is 2 orders of magnitude smaller than those from the Arrhenius and Hendrickson–Bray fits. This difference arises because the VTF model captures ion transport coupled to the segmental motion of the polymer. In this context, ion mobility is more significantly hindered by local structural dynamics. Furthermore, solvent processing of the membranes increases the onset temperature for the transition to Arrhenius behavior compared to dry processing (Figure 2d). This difference suggests variations in the microstructural uniformity of the HSE and DHSE. The prolonged VTF behavior indicates a less uniform dispersion of the filler particles in the DHSE, leading to large PEO bulk regions devoid of filler particles. These observations are also consistent with the DSC measurements (Figure S2), which show an additional melting point at a lower temperature in the DHSE. This implies that the filler does not integrate uniformly into the polymer matrix, resulting in a microstructure that varies in PEO crystallinity and the distribution of Li_6_PS_5_Cl particles.

**Figure 3:**
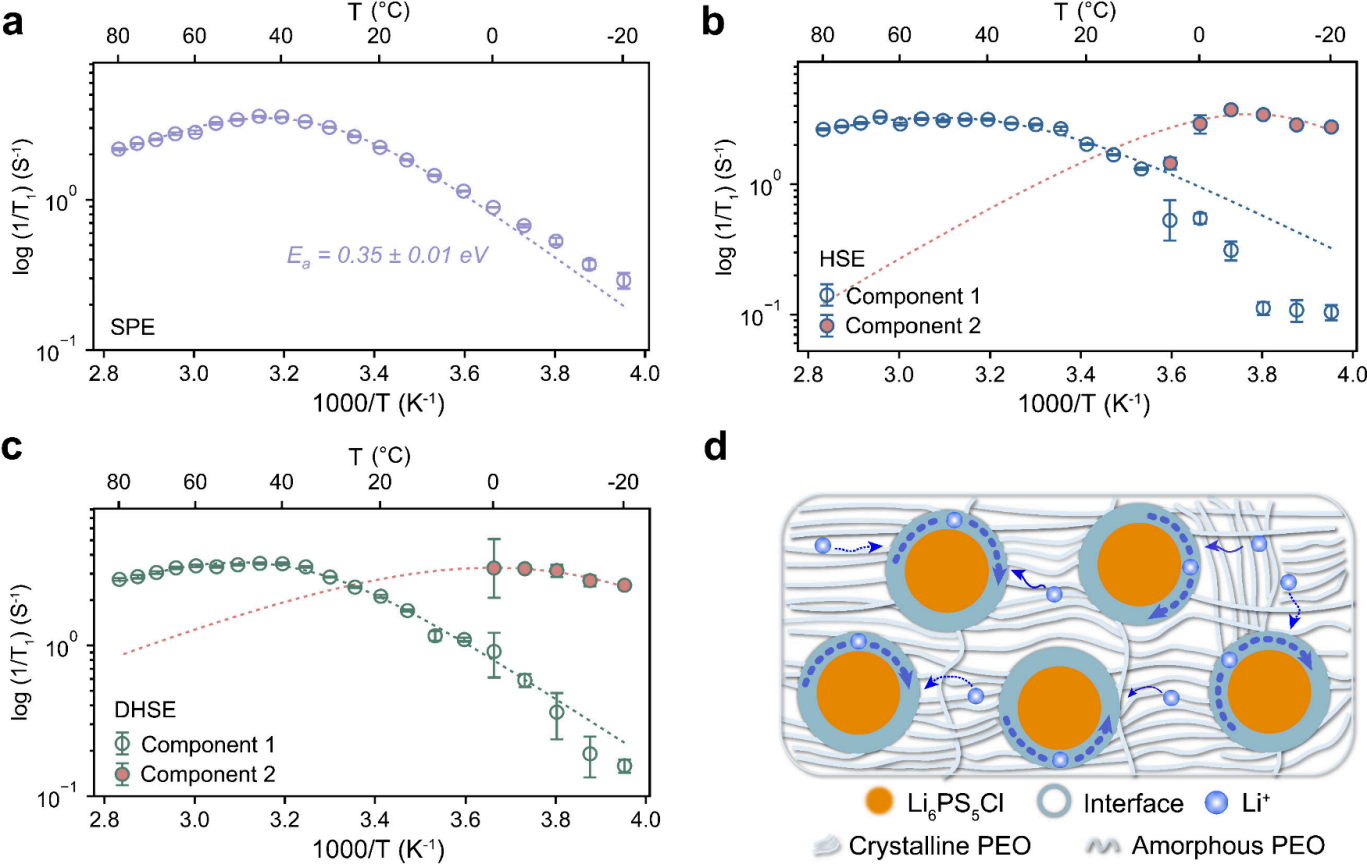
Extending insight into local Li-ion dynamics with ^7^Li *T*_1_ relaxometry measurements. ^7^Li relaxation rates measured for (a) the SPE, (b) the HSE, and (c) the DHSE. The fit in (a) represents a modified Bloembergen–Purcell–Pound (BPP) type spectral density, while the fits in (b) and (c) use the Richards spectral density model for two-dimensional (2D) diffusion, showing both single and two-component regions (see details in Supplementary Text 3). (d) Schematic showing the Li-ion diffusion pathways within the (D)HSE at low temperatures, with the polymer–filler interfaces gaining more Li-ions during the cooling process, leading to a fast Li-ion diffusion pathways along the interfaces at lower temperatures.

To further investigate the impact of fillers and processing methods on the dynamic coupling of Li-ions and polymer chains at local scales, spin–lattice relaxation (SLR) analysis at various temperatures was applied. This provides deeper insight into how local interactions fluctuate at frequencies that cause the probed Li nuclei to relax longitudinally. Relaxation rates (1/*T*_1_, *T*_1_: spin–lattice relaxation time) are sensitive to motion on a time scale comparable to the inverse of the NMR Larmor frequency (116.6 MHz for this study), i.e., several nanoseconds. Hence, SLR focuses on more localized motion compared with the previous line-width analysis. Both techniques probe local Li-ion motion at comparable length scales (Å to nm) but provide complementary information: line widths reflect motional averaging and structural heterogeneity, while *T*_1_ relaxometry probes local ion dynamics over time. Figure 3a–c shows the relaxation rates measured at increasing temperatures for the SPE, HSE, and DHSE respectively. The relaxation rates for the SPE can be fit with the modified Bloembergen–Purcell–Pound (BPP) spectral density function,^47^ which exhibits a broad peak with a maximum within the thermal window of 40 to 45 °C (see details in Supplementary Text 3). At this maximum, the Li hopping frequency is of the same order as the Larmor frequency. The BPP fit gives an *E*_a_ value of 0.35 ± 0.01 eV, which is much smaller than the *E*_a_ obtained from the Hendrickson–Bray fit for the line-width measurements (Figure 2a, 0.60 ± 0.03 eV). This difference arises as *T*_1_ measurements capture short-range Li-ion dynamics, whereas line-width analysis reflects environmental inhomogeneities and constraints on broader ionic motion.

The relaxation rates of the (D)HSE show a similar trend to the SPE when the temperature is ≥10 °C (Figure 3b,c). However, a separate Li-ion relaxation process from the polymer phase arises when the temperature ≤5 °C, as depicted by the filled circles (denoted as Component 2). The relaxation rate of the newly emerging component differs from that of the primary PEO component (denoted as Component 1) due to its considerably shorter relaxation times, and it is not identifiable in the pure polymer electrolyte system shown in Figure 3a. An example of the individual *T*_1_ fit at 0 °C is shown in Figure S6. The HSE and DHSE yield the best fits using a biexponential model, while the second exponential fit for the SPE data fails, indicating the presence of only one component. This secondary relaxation mechanism, nested within the LiTFSI-PEO peak, suggests a variant of EO-Li coordination with shorter relaxation times that are unaffected by the mobility of the polymer chains. Correlating these observations with the Abragam model analysis shown in Figure 2c,d suggests that a concentrated salt phase develops at the polymer–filler interfaces, as this feature is not present in the SPE sample. This is further supported by the lower *E*_a_ of the D(HSE) compared to that of the SPE in the low-temperature region, as determined from the VTF fit in the Abragam model analysis (Table S1). As depicted in Figure 3d, Li-ions preferentially move along the organic/inorganic interface with high salt concentration, which occurs uniquely at low temperatures in the HSEs. Comparing the two processing methods, notable differences were observed, especially at temperatures ≤10 °C. The trend line for Component 1 in the DHSE shows a more linear behavior compared to that of the HSE, indicating that the Li-ion diffusion in this phase resembles that observed in the SPE more closely. This divergence can be attributed to filler aggregation in the DHSE, leading to larger regions of the polymer phase lacking filler particles, thus locally behaving similarly to the SPE. Moreover, these agglomerates result in reduced polymer–filler interactions due to less contact surface area.

The modified BPP spectral density fit for the SPE indicates a reasonable fit to a single relaxation mechanism. However, this model is not feasible for fitting the relaxation data of the (D)HSE, as the appearance of Component 2 causes substantial deviations. Comparison with the τ_c_ data from the line-width measurements (Figure 2c,d) also shows a change in diffusion behavior at around 5 to 10 °C. This indicates the need for a different spectral density function to accurately fit the data. In this context, we attempted to fit Components 1 and 2 individually using the Richards spectral density function for two-dimensional (2D) diffusion. This provides a better fit to the measured data (see details in Supplementary Text 3),^48^ although the deviation for the HSE in Component 1 remains large below 10 °C. This indicates that the influence of the filler on the local dynamics of the polymer phase is more pronounced at and below this temperature. Any quantitative analysis for the (D)HSE relaxation rate curves is challenging due to the limited number of data points and the less defined maximum in the relaxation rate curves compared to the SPE. However, these observations clearly show a second relaxation process within the polymer matrix induced by filler addition. The absence of Component 2 in the SPE at low temperatures, along with the lower-temperature onset of the Richards-type behavior in the HSE compared to the BPP-type relaxation in the SPE, suggests 2D diffusion of Li-ions both within/between the polymer segments and along the polymer–filler interfaces (Figure 3d). The observation of the fast local dynamics of Li-ions from this second component could be leveraged to better understand and expand the operating temperature range of HSEs.

## Polymer Structure and Dynamics

**Figure 4:**
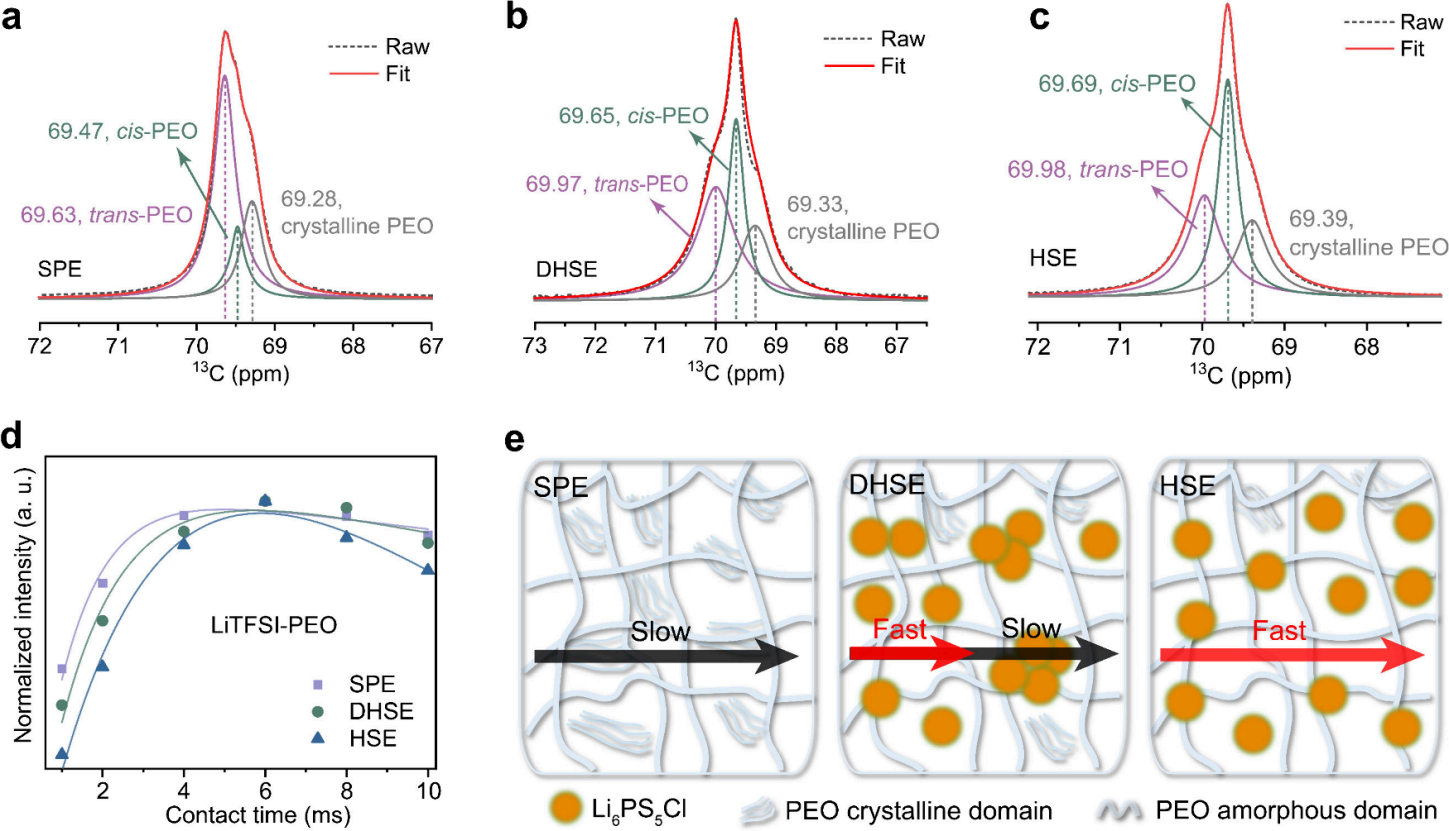
Characterizing the polymer conformation and dynamics. Fitting of the high power decoupling (hpdec) one-pulse ^13^C MAS solid-state NMR spectra for (a) the SPE, (b) the DHSE, and (c) the HSE. (d) Integrated intensities and the corresponding fits extracted from 1D ^7^Li (^1^H → ^7^Li) CP MAS spectra obtained for the SPE, DHSE, and HSE, expressed in arbitrary units (a.u.), with a focus on the LiTFSI-PEO environment (individual spectra at contact times ranging from 0.05 to 10 ms are shown in Figure S10; see fitting details in Supplementary Text 4). (e) Schematic showing the Li-ion transport modes in the SPE, DHSE, and HSE.

Given the distinctive differences in Li-ion dynamics between the SPE and HSEs, a detailed study of the polymer conformation was conducted by using room-temperature ^13^C NMR spectra to investigate the impact of the Li_6_PS_5_Cl filler and the processing method on the bulk structure. Deconvolution of the ^13^C spectra reveals that the ^13^C environments are dominated by *trans*-PEO, *cis*-PEO, and crystalline PEO in both the SPE and (D)HSE (Figure 4a–c).^49^ The slight downfield shift (to a higher ppm value) of the ^13^C chemical shift in the (D)HSE is attributed to enhanced polymer chain mobility, as evidenced by the normalized peak intensity in Figure S7. The crystalline phase ratios in the SPE, DHSE, and HSE are 30%, 25%, and 22%, respectively. This occurs since the addition of fillers increases the amorphous region of the polymer phase, leading to increased Li-ion coordination with EO groups and a consequent deshielding effect on the ^13^C environment. The SPE is predominantly composed of *trans*-PEO, whereas the HSE is dominated by *cis*-PEO, with the DHSE lying between the two. For the oxygen atoms adjacent to the *cis*-PEO carbon atoms, the shorter distance and smaller steric hindrance facilitate the coordination of Li-ions with these oxygens, as depicted in Figure S8. This enhanced coordination improves the Li-ion conductivity of the polymer chains, which corresponds to increased ionic conductivity and improved local Li-ion dynamics in the (D)HSE, as discussed in previous sections.

The structural change also coincides with alterations in polymer dynamics, as evidenced by the faster proton relaxation rates detected in the HSE from the ^1^H SLR measurements (Figure S9). This could be explained by an inherently higher mobility of the amorphous phase resulting from the addition of fillers. In addition, 1D ^7^Li (^1^H → ^7^Li) cross-polarization (CP) MAS experiments were conducted to distinguish local ^1^H and ^7^Li dynamics (Figure S10). In these experiments, magnetization from the abundant protons (^1^H) is transferred to nearby ^7^Li nuclei during a defined contact time, enabling the analysis of ^1^H dynamics. Focusing on the sharp LiTFSI-PEO peak, the buildup of the peak intensity can be fitted using the characteristic proton spin–lattice relaxation time in the rotating frame (*T*_1ρH_) values (fitting details in Supplementary Text 4). Among the three samples, the HSE displays a notably faster cross-relaxation time (Figure 4d and Table S2), suggesting a more mobile proton environment. This variation is also reflected in the *T*_1ρH_ values, as presented in Table S2. Despite the improvement in polymer dynamics with the addition of fillers, noticeable differences remain between the two processing methods. The proton dynamics in the DHSE are more similar to those in the SPE due to the inhomogeneous particle distribution, as shown in Figure 4d.

The impact of Li_6_PS_5_Cl fillers and the processing method on the polymer structure and dynamics in the PEO-based electrolytes is summarized in Figure 4e. In a typical PEO-LiTFSI SPE system, the high crystallinity of the polymer chains impedes efficient Li-ion transport. Introducing Li_6_PS_5_Cl fillers into the SPE system through solvent processing has proven effective in improving Li-ion conduction by reducing the crystallinity of the polymer and altering the polymer chain conformation. In contrast, the electrolytes produced by dry processing resulted in an inhomogeneous membrane characterized by phase separation and discontinuous Li-ion transport channels. This underscores the importance of uniformity to create continuous Li-ion pathways when designing hybrid electrolytes.

## Compatibility toward Li-Metal Anodes

**Figure 5:**
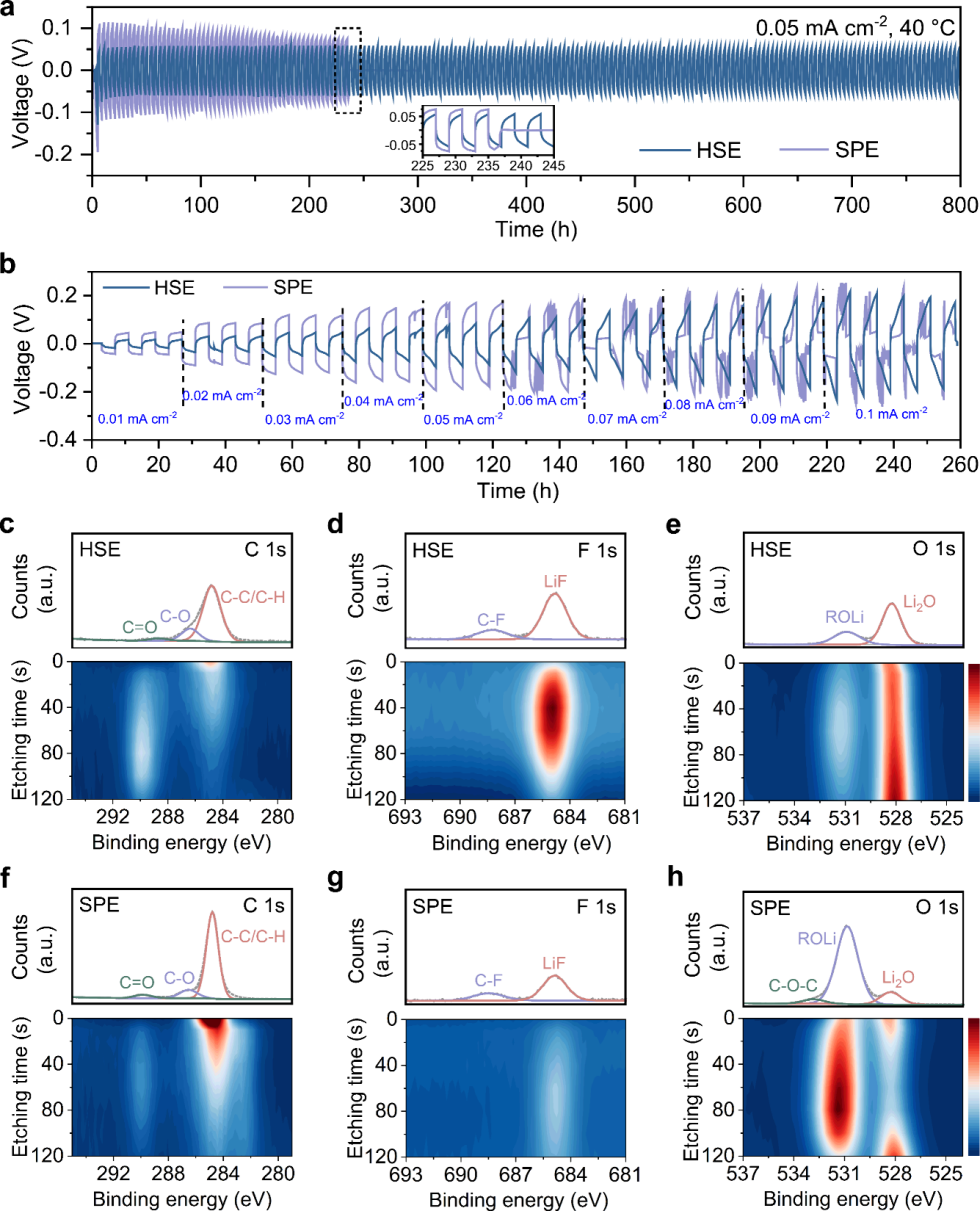
Electrochemical performance and interfacial properties with Li-metal. (a) Galvanostatic voltage profiles of the Li||Li symmetric cells measured at 0.05 mA cm^–2^ and 40 °C (0.05 mAh cm^–2^, with inserts showing the voltage profiles at ∼235 h). (b) Plating and stripping curves of the Li||Li symmetric cells measured at variable current densities and 40 °C. Depth-profiling XPS measurements of C 1s, F 1s, and O 1s for the Li-metal anode cycled with (c–e) the HSE and (f–h) the SPE. Each plot comprises two individual figures, i.e., the spectrum before etching (up) and depth profile (down). The dashed lines represent the raw data and the continuous lines are from the fits. The color bar indicates the intensity from low to high from bottom to top. The Li-metal electrodes were obtained by disassembling the Li||Li symmetric cells cycled with the SPE or HSE electrolytes at 0.05 mA cm^–1^ for 10 cycles (0.05 mAh cm^–1^, 40 °C).

Building on the understanding of Li-ion dynamics and polymer conformation in the HSE, the compatibility of the PEO-based electrolytes with and without Li_6_PS_5_Cl fillers toward Li-metal anode has been investigated using Li||Li symmetric cells. During long-term cycling at 0.05 mA cm^–2^ and 40 °C (Figure 5a), the cell cycled with the SPE consistently shows a higher overpotential compared to the HSE, which is ascribed to insufficient Li-ion conductivity of the SPE. In contrast, the cell using the HSE maintained a stable overpotential at ∼50 mV and remained stable for over 800 h, whereas the cell using the SPE failed at ∼237 h (Figure 5a, insert). When increasing the current density, the Li|HSE|Li cell exhibits a much more stable overpotential, whereas the Li|SPE|Li cell already fails at an overpotential as low as 0.05 mA cm^–2^ (Figure 5b). The improved performance is attributed to the addition of Li_6_PS_5_Cl fillers, which enhance both the ionic conductivity and the mechanical strength of the SPE, thereby effectively suppressing Li dendrites.

To further understand the enhanced interfacial stability of the HSE toward Li-metal, both the pristine electrolytes and the solid electrolyte interphase (SEI) formed on the Li anode disk after cycling were investigated using depth-profiling X-ray photoelectron spectroscopy (XPS). Figure 5 panels c–h depict the depth-dependent evolution of the C 1s, F 1s, and O 1s XPS spectra for both the SPE and HSE. Within the C 1s spectra (Figure 5c,f), the peak corresponding to C–C/C–H is attributed to the ether groups of the PEO residues, while the smaller peaks of C–O and C=O arise from the decomposition products of PEO.^50,51^ It is evident that the SEI formed with the SPE exhibits a more pronounced organic outer layer on its surface, as indicated by the more intense C–C/C–H peak. In the F 1s spectra (Figure 5d,g), the LiF peak and the C–F peak originate from the decomposition and residuals of LiTFSI.^52−54^ The evolution of the LiF peak intensity contrast between these two samples indicates the formation of a LiF-rich SEI layer of the Li-metal cycled with the HSE. Tracking the relative spectral contribution of the O 1s species as a function of the etching time (Figure 5e,h) indicates that the predominant presence of Li_2_O in the SEI layer formed with the HSE.^55,56^ In contrast, the SEI layer formed with the SPE shows a prevalent distribution of the organic ROCO_2_Li species.^57−59^ When correlating the observed SEI compositions with the surface properties of the pristine electrolytes, the SPE exhibits a higher concentration of the PEO-LiTFSI phase and LiF on its surface, as indicated by the more pronounced PEO characteristic peaks and LiF peak in Figure S11. This is likely due to the presence of a filler in the HSE, which weakens the signal from the polymer phase.

Although the Li-ion exchange between the PEO and Li_6_PS_5_Cl phases is not as significant as in the PEO–garnet composite system, where it minimizes charge gradients at the electrolyte/electrode interfaces to facilitate homogeneous Li electrodeposition,^13,60^ the observed difference in SEI composition suggests that the presence of Li_6_PS_5_Cl alters the decomposition sequence of LiTFSI at the Li-metal surface. This alteration promotes the formation of inorganic SEI components such as LiF and Li_2_O, which results in an inorganic-rich SEI that is Li-ion conductive and more effective in suppressing dendrite propagation.^61^ X-ray diffraction (XRD) analysis confirms the retention of the Li_6_PS_5_Cl crystalline phase, indicating its structural stability within the PEO matrix (Figure S12). This stability also contributes to the enhanced ionic conductivity and mechanical strength of the HSE, which work collaboratively to reduce polarization and distribute local stress more efficiently.

In summary, incorporating Li_6_PS_5_Cl inorganic fillers into the PEO-LiTFSI polymer electrolyte, especially when processed using a solvent-assisted method, considerably improves the ionic conductivity of the SPE. The underlying mechanism is elucidated through an analysis of local Li-ion dynamics, leveraging NMR’s capability to provide insight in the dynamic and structural properties. The line-width analysis indicates that adding filler suppresses the crystallization of the polymer phase as the temperature decreases. The activation energies obtained from the Hendrickson–Bray and Abragam model fits of the line widths suggest enhanced local Li-ion mobility in the HSEs compared to the SPE. The temperature-dependent correlation time, τ_c_, further captures the characteristic phase transitions of the PEO phase, which reveals typical VTF behavior in the intermediate temperature range and Arrhenius behavior in the fully crystallized state. At low temperatures, the second *T*_1_ relaxation component in the HSEs indicates two distinct dynamic modes within the polymer matrix induced by the presence of the filler. The 2D Richards spectral density function fitting indicates that the Li-ion transport occurs along the salt-rich polymer–filler interfaces. Despite the increased local Li-ion mobility from filler addition, morphological inhomogeneities may counteract this effect, as evidenced by the lower conductivity and Li-ion dynamics observed in the dry-processed electrolyte compared to its solvent-processed counterpart. The improvement in local Li-ion dynamics with filler incorporation correlates with increased segmental motion and a more conductive polymer conformation. Finally, the formation of an inorganic-rich SEI layer at the Li/HSE interface can be adopted in designing high-performance HSEs for enabling Li-metal anodes. The present work provides an in-depth understanding of local ion conduction and polymer–filler interactions in the PEO-sulfide hybrid electrolyte system, with potential applications to other electrolyte systems.

## Data Availability

All data needed to evaluate the conclusions in the paper are present in the paper and/or the Supporting Information.

## Supporting Information Available

Experimental procedures and supplementary texts (Hendrickson–Bray’s phenomenological equation, Abragam model, spectral density fitting, cross-polarization), SEM image, DSC, one-pulse ^7^Li/^1^H NMR spectra, ^7^Li line width, ^7^Li *T*_1_ relaxometry, normalized peak intensity of the one-pulse ^13^C NMR spectra, carbon conformation diagram, ^1^H *T*_1_ relaxometry measurements, 1D ^7^Li CP MAS NMR spectra, XPS spectra, XRD pattern, and supplementary tables (activation energies, fitting results and parameters) (PDF)
