# Supplementary material for: Revealing Local Diffusion Dynamics in Hybrid Solid Electrolytes
Source: ACS Energy Lett. 2025 Mar 17;10(4):1762–71. doi: 10.1021/acsenergylett.5c00214 (PMC11998072; doi:10.1021/acsenergylett.5c00214)
Supplement: Supplementary file 1 — nz5c00214_si_001.pdf [file nz5c00214_si_001.pdf]

Supplementing Information

Revealing Local Diffusion Dynamics in Hybrid Solid Electrolytes

Shengnan Zhang[1], Leon Felix Mueller[1], Laurence Macray[1], Marnix Wagemaker[1], Lars J. Bannenberg[1*], Swapna Ganapathy[1*]

[1]Section Storage of Electrochemical Energy, Radiation Science and Technology, Faculty of Applied Sciences, Delft University of Technology, Mekelweg 15, 2629 JB, Delft, The Netherlands.

[*]Corresponding authors: l.j.bannenberg@tudelft.nl and s.ganapathy@tudelft.nl

Methods and Materials

Preparation of PEO-based solid-state electrolytes

For both the SPE and (D)HSE, 384 mg PEO powder (M_w = 600, 000 g/mol, Sigma-Aldrich) and 140 mg LiTFSI (Sigma-Aldrich) were used. SPE was prepared by mixing PEO and LiTFSI in 7 mL acetonitrile (Sigma-Aldrich) and stirring for 24 hours. The obtained homogenized slurry was cast onto a Teflon plate and dried in the glove box at room temperature for 24 hours, followed by additional drying under vacuum for 48 hours. HSE was fabricated by first grinding the PEO, LiTFSI, and 52.4 mg Li_6PS_5Cl (10 wt%, NEI Corporation) in a mortar. The 10 wt% loading of Li_6PS_5Cl corresponds to 8 vol%, calculated based on the density (ρ) of each component: ρ_{LPSC} (Li_6PS_5Cl) = 1.6 g/cm^3; ρ_{PEO}= 1.2 g/cm^3; and ρ_{LiTFSI}= 1.3 g/cm^3, with an EO:Li ratio of18:1. The obtained mixture was then added to 7 ml of acetonitrile and stirred for 24 hours. The mixer used is an IKA Magnetic Stirrers RCT basic, operated at a stirring speed of 400-550 rpm at room temperature. The subsequent casting and drying procedures are the same as those used for the SPE.

The DHSE films were prepared using a hot-press method. The aforementioned amount of LiTFSI and Li_6PS_5Cl were first mixed by grinding in a mortar for over 15 minutes, then the PEO powder was added gradually in approximately 50 mg increments, with around 2 minutes of grinding between each addition. After all the PEO powder has been added, grind the mixture for over 15 minutes until a homogeneous dough forms. The resulting dough is then sandwiched between two polytetrafluoroethylene (PTFE) sheets, with a 150 μm spacer in between to control the thickness, and placed in a two-plate stainless steel assembly. This assembly is then heated on a hot plate set to 110°C. Once the target temperature is reached, it is held at that temperature for 10 minutes. Afterwards, the assembly is removed and compressed under a hydraulic press at 35 bar. Following compression, the dough is flattened into a membrane and then folded to improve mixing. The process of heating, compressing, and folding is repeated three times to ensure uniformity. Finally, the assembly is cooled under continuous compression for 12 hours. This extended cooling period ensures that the membrane cools gradually and uniformly, reducing exposure to external temperatures.

Material characterizations

For scanning electron microscope (SEM) imaging, the samples were transferred into an SEM (JEOL JSM-6010LA) machine using an airtight Argon-filled sample holder. The images were acquired with a 10 kV accelerating voltage (secondary electron). The SEM image of Li_6PS_5Cl particles was analyzed using the ImageJ software to calculate the particle size distribution. The equivalent particle diameter (d) was calculated from the projected two-dimensional area (A) of each particle using the formula: $d = \sqrt{4A/\pi}$. Bragg-Brentano X-ray diffraction (XRD) measurements were conducted on an X'Pert Pro X-ray diffractometer (PANalytical) with Cu Kα radiation (λ_1 = 1.540598 Å and λ_2 = 1.544426 Å, at 45 kV and 40 mA) using an in-house air-tight holder. Differential scanning calorimetry (DSC) measurements

were performed at 10° min^{-1} steps using a commercial TA-Q2000 DSC calorimeter (TA instruments). X-ray photoelectron spectroscopy (XPS) measurements were carried out with a Thermo Fisher K-Alpha spectrometer. The samples were transferred to the XPS machine under vacuum using an air-tight sample holder. The spectrometer is equipped with a focused monochromatic Al kα source (1486.6 eV) anode operating at 36 W (12 kV, 3 mA), and a flood gun operating at 1 V, 100 µA. The base pressure of the analysis chamber was approximately 2×10^{-9} mbar and the spot size was approximately 800×400 um^2. For the detailed scan collected before etching, a pass energy of 50 eV was used. Depth profiling was performed with an Ar^+ ion gun operated at 3 kV, corresponding to about 0.5 nm s^{-1} as calibrated on Ta_2O_5 and a pass energy of 200 eV was used. In the analysis, the binding energy was corrected for the charge shift relative to the primary C 1s hydrocarbon peak at $BE = 284.8$ eV. For each sample, at least 3 points were measured which showed consistent results. The data were fitted using 70% Gaussian and 30% Lorentzian line shapes (weighted least-squares fitting method) and nonlinear Shirley-type background using the Thermo Fisher Avantage software.

Batteries assembly and electrochemical measurements

The ionic conductivity of the SPE and (D)HSE were calculated based on electrochemical impedance spectroscopy (EIS) data obtained using an electrochemical station (Autolab PGSTAT302N). The measurements were conducted from 10 MHz to 1 kHz with a sinusoidal signal (V_{rms} = 10 mV). The electrolytes were sandwiched between two stainless steels (SS) in coin cells, and each test temperature was maintained for more than 30 minutes to ensure thermal equilibrium. The EIS data was fitted using an Equivalent Circuit (EC) model that included R_S as the series resistance, R_b as the bulk electrolyte resistance, and CPE_b as a Constant Phase Element (CPE) representing the bulk capacitance of the electrolyte. Additionally, CPE_{int} was introduced to account for the capacitance associated with the blocking electrodes at low frequencies. Subsequently, the bulk resistance (R_b) and ionic conductivity (σ) were determined using the formula $\sigma = d/(R_b \times A)$, with d representing the thickness of the electrolytes and A denoting the area of the electrolytes in contact with the stainless steels. The galvanostatic charge-discharge tests of the Li||Li symmetrical cells were performed on a Maccor 4000 battery cycler at 40 ℃.

Solid-state NMR measurements

The temperature-dependent ^{7}Li line-width and T_1 relaxometry measurements were conducted using a Bruker Avance 300 MHz wide-bore spectrometer (B_0 = 7.1 T) equipped with an NEO console, operating at frequencies of 116.64 MHz for ^{7}Li. A 90° pulse optimization was performed for each sample, and the probe was matched and tuned at every other temperature increment. The protocol began at ambient temperature (25 °C) and involved temperature decrements of 5 °C down to -20 °C. Before each measurement, the system was equilibrated for at least 10 minutes to ensure thermal stability. The T_1 relaxation constants were measured using a saturation recovery experiment. The one-pulse ^{7}Li

measurements were extended to lower temperatures (-55 °C). Post-completion of the low-temperature series, the system was gradually returned to room temperature. High-temperature experiments were then performed, with the temperature progressively increased by 5 °C up to a maximum of 80 °C for high temperature experiments.

Additional magic angle spinning (MAS) solid-state NMR measurements were performed on Bruker Ascend 500 MHz magnet (B_0 = 11.7 T) at room temperature, also equipped with an NEO console, operating at frequencies of 194.37 MHz for ^{7}Li, 500.130 MHz for ^{1}H, and 125.758 MHz for ^{13}C. One-pulse ^{7}Li and ^{1}H experiments were performed with 90° pulse lengths of 3.25 µs and 4 µs. A recycle delay of about four times T_1 was used for each nuclei, where the T_1 was determined using saturation recovery experiments. For 1D ^{7}Li (^{1}H→^{7}Li) CP MAS experiments, a radio frequency (r.f.) field strengths of 81 kHz and various contact times were applied, the r.f. field amplitude of ^{1}H during CP experiments was ramped from 70 to 100% and 128 scans were acquired for each sample with a recycle delay of 7 s. High power decoupling (hpdec) one-pulse ^{13}C measurements were conducted with a 90° pulse length of 5.95 µs and an recycle delay of 1 s, and the data were fitted using MestReNova 11.

All measurements were conducted using a Bruker three-channel MAS 4 mm direct variable temperature (DVT) probe. The chemical shifts of ^{7}Li were referenced with respect to a 0.1 M LiCl solution (0 ppm), while the chemical shifts of ^{1}H and ^{13}C were referenced to solid adamantane (^{1}H at 1.81 ppm and ^{13}C at 38.48 ppm). Individual samples were prepared by cutting the electrolyte membranes into small pieces, which were then transferred into 4 mm zirconia rotors and sealed with a Vespel cap. A MAS frequency of 10 kHz was used for the MAS measurements. For the ^{7}Li line-width and T_1 relaxometry measurements, the cap was modified by filing down its drive fins to prevent rotation, thus enabling static measurements.

Supplementary Texts

Text 1: Hendrickson Bray's phenomenological equation

This approach is based on the paper by J. R. Hendrickson and P. Bray,[1] which employs a phenomenological method to NMR motional narrowing. The model considers the system to consist of two states, the first component to consist of thermally non-excited ions, each characterized by a spin-spin relaxation time T_{2A} related to a broad line-width of A:

$$T_{2A} = \frac{1}{A} (1)$$

and the second component is that of thermally excited ions with a spin-spin relaxation time T_{2B} and a narrow line-width of B:

$$T_{2B} = \frac{1}{B} (2)$$

Experimentally, only one single NMR line with a line-width ω is observed, which is attributed to the rapid exchange between the two states. Consequently, the line-width is a combination of both fractions f_A and f_B, each weighted by their respective T_2 values:

$$T_2 = f_A T_{2A} + f_B T_{2B} = \frac{1}{\omega} (3)$$

The total fraction of ions is unity, and the fraction of thermally activated ions f_B can be approximated using the Arrhenius equation, which is given by:

$$f_B = \exp\left(- \frac{E_a}{kT} \right) (4)$$

where E_a is the activation energy, k is Boltzmann's constant and T is the absolute temperature.

Combining equations (1), (2), (3), and (4), the equation for the temperature-dependent line-width $\omega(T)$ becomes:

$$\omega(T) = A\left[\left(1 + \left(\left(\frac{A}{B} - 1\right) exp\left(- \frac{E_a}{kT}\right)\right)\right)\right]^{-1} + (5)$$

where D is the correction factor.

Text 2: Abragam model

The rate of fluctuations in the local field of a spin can be described by a characteristic rotational correlation time τ_c. Fast fluctuations, which average out interactions, are given with respect to the instantaneous Larmor precession (ω_0) in the local field, and are typically categorized as:[2]

$$\omega_0\tau_c \ll (6)$$

The product of the Larmor frequency and correlation time should be much less than 1 for the fast regime. If this inequality is true, there will be appreciable narrowing. Conversely, when ω_0 τ_c is large, the NMR signals are influenced by slower motions, and the regime is often termed the "slow exchange" regime. Qualitatively the line-width can be defined as the inverse of the time (*t*), after which spins precessing in their respective local field, which were initially in phase, are now out of phase:

$$t \cong \frac{1}{v}(7)$$

where v is the line-width (usually characterized by full width half maximum, FWMH) at a given temperature. Motional narrowing starts when the rate of fluctuation, described by the correlation time τ_c, is fast enough to start averaging out interactions, thus:

$$\omega_0 \ll \frac{1}{\tau_c}(8)$$

From this assumption, an estimation for the correlation time at any line-width can be obtained by the following relationship:[2]

$$\tau_c = \frac{\alpha}{v}\tan\left[\frac{\pi}{2}\left(\frac{v}{v_{rigid}}\right)^2\right](9)$$

where v_{rigid} is the FWHM of the rigid phase, or the FWHM measured at the lowest temperate in this case, and α is a constant close to 1. It should be noted that when $v \approx v_{rigid}$, the term tan $\left[\frac{\pi}{2}\left(\frac{v}{v_{rigid}}\right)\right]^2$ becomes very large, which causes significant uncertainties in τ_c. Therefore, R_{rigid} is excluded from detailed analysis in **Figure 2c,d**. In some cases, τ_c is a thermally activated jump process described by Arrhenius:

$$\tau_c = \tau_0\exp\left(\frac{E_a}{kT}\right)(10)$$

where τ_0 is the pre-exponential constant, E_a is the activation energy, k is Boltzmann's constant and T is the absolute temperature. However, in polymers, diffusion processes are not governed by a strict Arrhenius behavior. The Vogel-Tamman-Flucher (VTF) equation is often employed to describe the conductivity behavior of materials approaching their glass transition temperature (T_g).[3] Hence, a modified equation is used to describe the correlation time:

$$\tau_c = \tau_0 \exp\left(\frac{A}{k(T - T_0)}\right) (11)$$

where τ_0 is the pre-exponential constant, A is the pseudo-activation energy, and T_0 is the ideal glass transition temperature. The VTF equation assumes that mobility is affected by free volume of the polymer, which exhibits a non-linear temperature dependence and diminishes to zero at T_0, where polymer chain motion ceases.

It is noteworthy that activation energies obtained from the VTF fit and the Arrhenius and Hendrickson Bray's models are not directly comparable, as they correspond to distinct physical behavior in the polymer phase under different temperature regions.[4] The Arrhenius model describes a transport mechanism in which cation motion is independent of the molecular dynamics of the polymer host. When the temperature-dependent Li-ion motion follows an Arrhenius relationship, Li-ion transport resembles that observed in ionic crystals, where ions migrate by jumping between adjacent vacant sites. In the Hendrickson Bray's model, overall Li-ion dynamics are captured by accounting for interactions from all phases present in the system: crystalline, semicrystalline, and amorphous phases, which collectively influence the observed line broadening. In contrast, the VTF model describes ion transport in systems exhibiting non-Arrhenius behavior, a characteristic often observed in solid polymer electrolytes. It highlights a strong inter-relation between ion conductivity and polymer segmental relaxation.[5] In this context, the activation energy reflects the temperature dependence of ion mobility, which is closely coupled with the segmental motion of the polymer. This coupling results in the characteristic curvature of the VTF behavior, where the activation energy derived from the VTF fit is typically lower, reflecting slow, cooperative ion dynamics, especially as the system approaches the melting temperature. The lower activation energy values obtained from the VTF model captures the physical processes governing ion transport in heterogeneous systems, where ion mobility is more significantly hindered by local structural dynamics.

Text 3: Spectral density fitting

Interpreting the shape of relaxation data and its implications for the motional processes of the probed Li-ions is not straightforward. The measured T_1 (spin-lattice relaxation time) needs to be linked to the correlation time τ_c of the probed nucleus in its environment, to determine the activation energy for the observed motional process. Spectral density fitting plays a pivotal role in understanding of the complex interplays. The spectral density function $J(\omega)$ is the Fourier transform (in the frequency domain) of the autocorrelation function $G(t)$ (in the time domain), which quantifies how the local magnetic field at one time correlates with its magnitude and orientation at a later point in time.[6,7] The spectral densities reveal patterns of spin fluctuations, and characterizing these patterns provides a deeper understanding of the system's dynamics.

Redfield equation

In a sample that is mostly dominated by dipolar relaxation, the spin-lattice relaxation rates can be described by the Redfield equation:[2]

$$\frac{1}{T_1} = K_D[J(\omega_0) + 4J(2\omega_0)](12)$$

This expresses the spin-lattice relaxation rate $1/T_1$ as a function of the spectral density function with a proportionality constant K_D, which is related to the strength of the dominant spin interaction, whether dipolar or quadrupolar. Spectral density fitting involves adjusting the parameters of a chosen model of $J(\omega_0)$ so that the model accurately reflects the experimentally measured relaxation times. This process enables us to infer details about the molecular motions that influence the relaxation behavior.

Bloembergen-Purcell- Pound (BPP) model

The simplest and most common spectral density model is the Bloembergen-Purcell-Pound (BPP) model. This model assumes isotropic random three-dimensional (3D) molecular motion with a single thermally activated correlation time τ_c.[7]

$$J_{BPP}\,(\omega_0, \tau_c) = \frac{2\tau_c}{1 + (\tau_c\omega_0)^2}(13)$$

where τ_c can be described with the Arrhenius equation or VTF equation. The position of the maxima or, where the relaxation rate is highest, is when $\omega\tau_c = 1$ (when the correlation time is the inverse of the Larmor frequency). The spectral density then has limiting values far away

from the maxima. At the low temperature limit ($\omega\tau_c \ll 1$) and high temperature limit ($\omega\tau_c \gg 1$), the spectral density reduced to a simpler form:

$$J_{BPP}(\omega_0, \tau_c) = \begin{cases} 2\tau_c, & \omega_0\tau_c \ll \\ 2/(\tau_c\omega_0{}^2), & \omega_0\tau_c \gg \end{cases} (14)$$

When substituting into equation (12) and plotting the natural log of ($1/T_1$) against ($1/k$T), the slopes of the low and high temperature regions correspond to $-E_a$ and $+E_a$, respectively. In more complex systems, deviations from this model often arise due to factors such as system disorder or strong Coulomb interactions among the moving ions, leading to asymmetrical slopes.[8] To account for this asymmetry, an asymmetry parameter β is introduced:

$$\beta = \frac{E_{a,\,LT}}{E_{a,\,HL}} + (15)$$

The overall spectral density is given by:

$$J_{\,BPP}(\omega_0, \tau_c\,) \propto \frac{\tau_c}{1 + (\omega_0\tau_c)^\beta} (16)$$

This is often referred to as the modified BPP model. The full expression that is fit to the relaxation rates reads as follows:

$$\frac{1}{T_1} = K_D\left[\frac{2\tau_c}{1 + (\omega_0\tau_c)^\beta} + \frac{4\tau_c}{1 + (2\omega_0\tau_c)^\beta}\right] (17)$$

Richards model

In some systems, diffusion may be restricted to two dimensions. Unlike the symmetric peaks observed with 3D diffusion-induced relaxation rates, two-dimensional (2D) diffusion exhibits an asymmetric profile, typically with a lower slope on the high-temperature side. This can occur in cases such as conduction along an interface or surface. To model such systems, Richards proposed an empirical expression that combines the two limiting cases for low and high temperatures:[9]

$$J_{2D(\omega_0,\,\tau_c)} \propto \begin{cases} \omega_0{}^{-2}\tau_c{}^{-1}, & \omega_0\tau_c \gg 1; \\ \tau_c \ln(1/\omega_0\tau_c), & \omega_0\tau_c \ll 1. \end{cases} (18)$$

combined into the empirical expression:

$$J_{2D}(\omega_0, \tau_c) \propto \tau_c \ln\left(1 + \frac{1}{(\omega_0\tau_c)^\beta}\right) (19)$$

Here, the parameter β is expected to be equal to 2 for following the BPP behavior. If this accurately approximates the underlying dynamics, the frequency and temperature dependencies are described by a single equation $J(\omega_0, \tau_c)$. This results in a slightly asymmetric rate peak, with the high-temperature slope being somewhat reduced compared to the low-temperature slope, which is the opposite of the adjusted BPP model.

The full expression that is fit to the relaxation rates reads as follows:

$$\frac{1}{T_1} = K_D\left[\tau_c \ln\left(1 + \frac{1}{(\omega_0\tau_c)^{\beta}}\right) + 4\tau_c \ln\left(1 + \frac{1}{(2\omega_0\tau_c)^{\beta}}\right)\right] (20)$$

Multiple correlation times

Electrolytes often exhibit multiple simultaneous diffusion processes within the system. In such systems, where different thermally activated processes have distinct rates and activation energies, these processes contribute additively, assuming they do not interfere with one another. Mathematically, this can be expressed as:

$$\frac{1}{T_{1,\,total}} = \frac{1}{T_{1,a}} + \frac{1}{T_{1,b}} + \cdots + \frac{1}{T_{1,N}} (21)$$

This means that it is possible to fit complex T_1 data as a sum of superimposed individual processes.

Text 4: Cross-polarization

Cross-polarization (CP) dynamics depend on the effective heteronuclear dipolar interaction ($T_{I-S} \approx 1/r^6$, where r is the distance between the nuclei) between ^{1}H and ^{7}Li, as well as on local mobilities that affect the relaxation of magnetization ($T_{1\rho}$ for both ^{7}Li and ^{1}H). A stronger effective dipolar interaction leads to a faster buildup while motions can reduce the effectiveness of transfer as well as enhancing the decay of magnetization if they are in the range of strength of the CP locking fields. The broad peaks observed near the main LiTFSI-PEO resonance in the spectra of HSE and DHSE correspond to the interfacial products that are in close proximity to the neighboring protons (**Figure S10**), consistent with our previous work.[10] Focusing on the main LiTFSI-PEO environment (in the dotted box), both the HSE and DHSE have much smaller cross-relaxation time (T_{H-Li}) than that for the SPE, with the DHSE showing slower cross-relaxation than the HSE. These differences are also reflected in their distinct proton $T_{1\rho}$ ($T_{1\rho H}$) values (**Table S2**). This effect is attributed to changes in the proton environment due to

the disruption of the PEO structure by fillers, which alters the proton dynamics on a time scale that does not affect the transfer rate but results in different $T_{1\rho H}$ values for both samples. This suggests that the polymer-filler interactions improve local proton dynamics, with this improvement closely associated with local morphological homogeneity.

Supplementary Figures

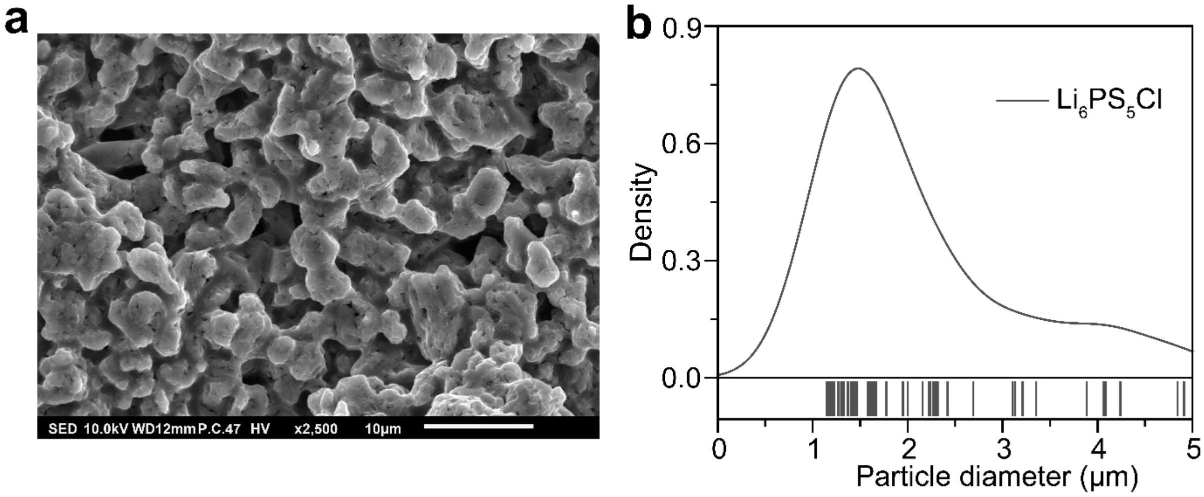

Figure S1. Characterization of the pristine Li_6PS_5Cl. (**a**) SEM image of the pristine Li_6PS_5Cl particles. (**b**) Size distribution of the pristine Li_6PS_5Cl particles, calculated from the SEM image in (**a**) using image analysis software ImageJ. The particle diameters were derived from the measured particle areas (50 particles in total), assuming an approximately circular shape.

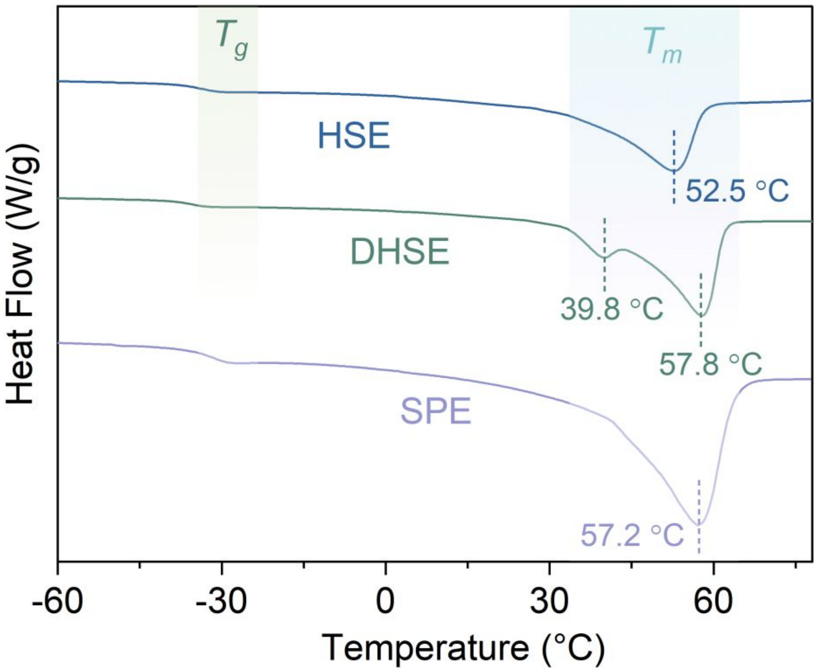

Figure S2. DSC measurements showing the heat flow of the SPE and (D)HSE. DSC curves of the SPE and (D)HSE under heating. The glass transition temperature (T_g) region and melting temperatures (T_m) region are highlighted. The DHSE shows an additional melting point at 39.8 °C, indicating that the filler does not integrate uniformly into the polymer matrix, which result in a microstructure with varying PEO crystallinity and inhomogeneous distribution of Li_6PS_5Cl particles.

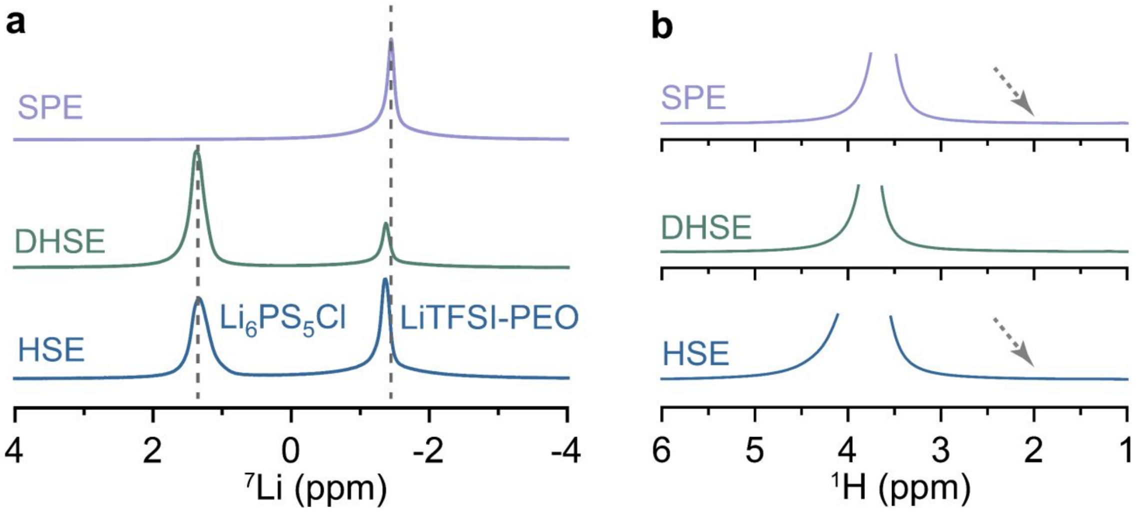

Figure S3. Characterization of the SPE and (D)HSE. (**a**) One-pulse ^{7}Li MAS NMR spectra of the SPE, DHSE, and HSE. The relatively low intensity of the LiTFSI-PEO environment in the DHSE is indicative of the sample heterogeneity, which is attributed to more Li_6PS_5Cl and less polymer phase in the rotor. Since the MAS measurement captures an average signal, the peak intensity reflects the absolute amount of each environment. (**b**) Zoom-in view of the 1D ^{1}H MAS NMR spectra in **Figure 1e**. The arrows indicate the ^{1}H chemical shift position corresponding to acetonitrile solvent,[11] where no signal is observed, confirming the absence of residual acetonitrile.

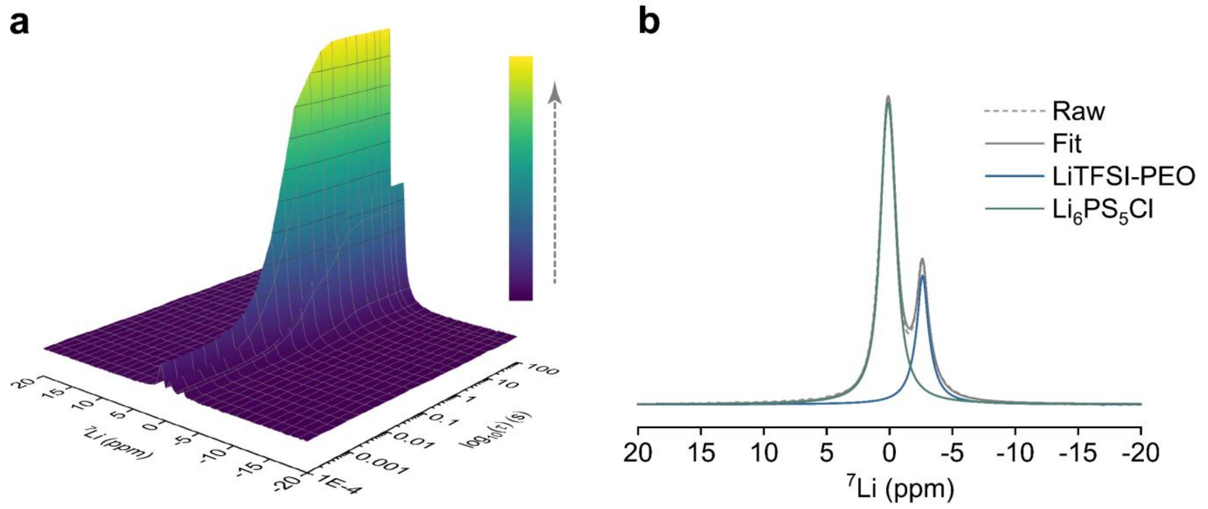

Figure S4. Peak deconvolution of the obtained ^7Li NMR spectra from the line-width and $\boldsymbol{T_1}$ relaxometry measurements for the (D)HSE. Using the T_1 measurement for the HSE at 30 °C as an example. Breaking down the spectrum into individual Lorentzian functions for each peak allows for a more accurate determination of both line-widths and amplitudes, effectively separating the contributions from the PEO and Li_6PS_5Cl phases. (**a**) 3D plot of the T_1 measurement before deconvolution, with the chemical shift in ppm on the x-axis, the logarithm of the recovery time (τ) on the y-axis, and the peak intensity on the z-axis. (**b**) The spectrum after deconvolution, showing the same T_1 measurement at $\tau = 100\ s$. The color bar indicates the intensity from low to high from bottom to top.

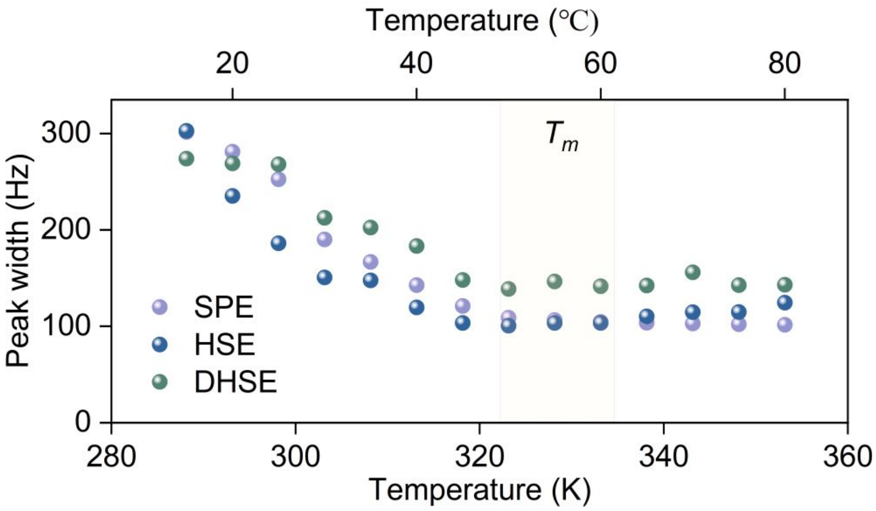

Figure S5. Probing local Li-ion dynamics using ^{7}Li line-width analysis. Zoomed-in view of the temperature-dependent ^{7}Li line-width measurements in **Figure 2a,b** for the SPE and (D)HSE, within a temperature range of 15 to 80 °C.

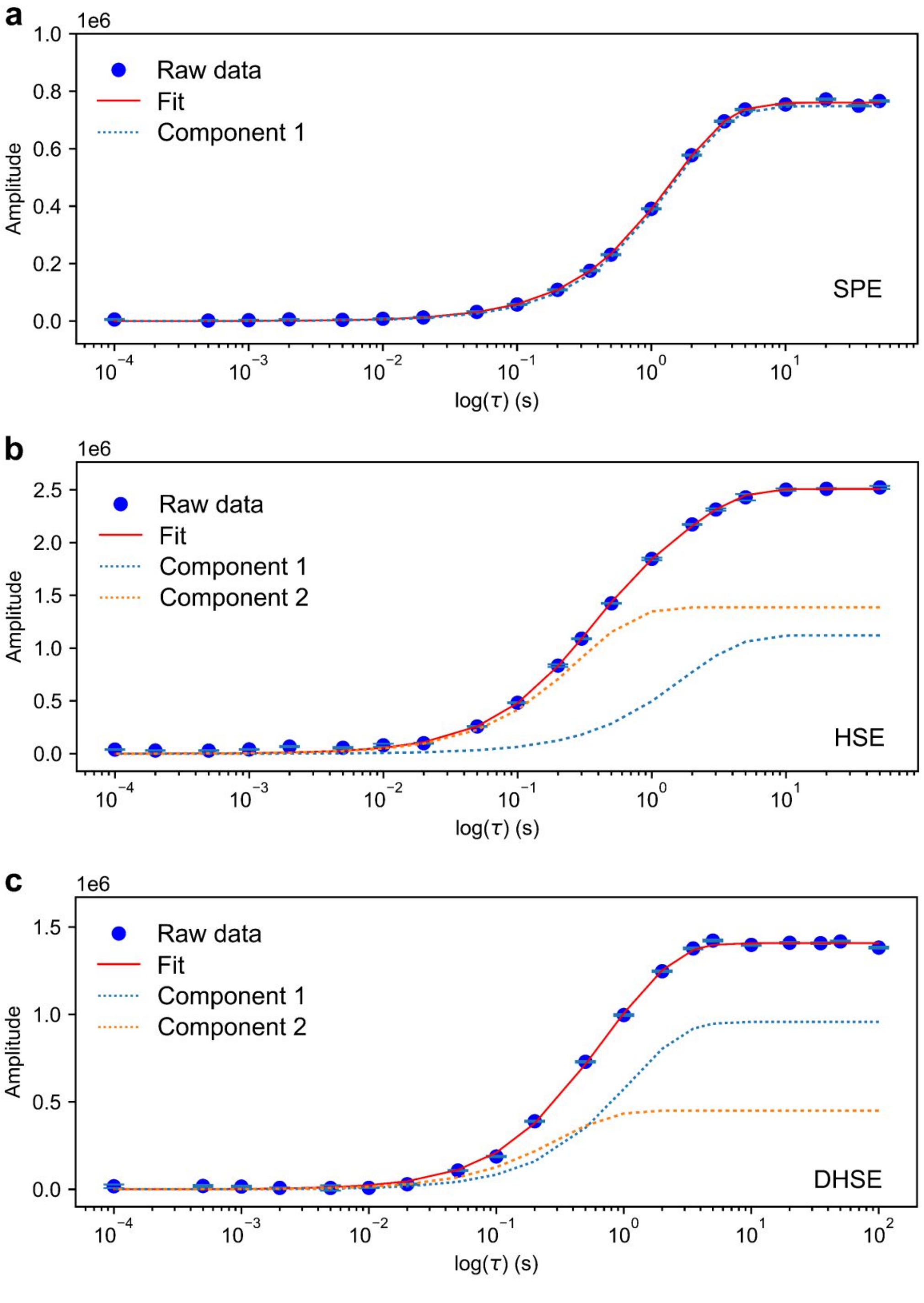

Figure S6. Probing local Li-ion dynamics through ^{7}Li T_1 relaxometry measurements. Bi-exponential recovery fit for (**a**) SPE, (**b**) HSE, and (**c**) DHSE. For all samples, the data were obtained at 0 °C, with τ representing the recovery time in the saturation recovery experiments[12] for the T_1 measurements. For the HSE and DHSE, optimal fits were achieved using a bi-exponential recovery model, where each component corresponds to distinct T_1 dynamics. In contrast, the fitting of the second component for the SPE data failed, indicating the presence of only one component.

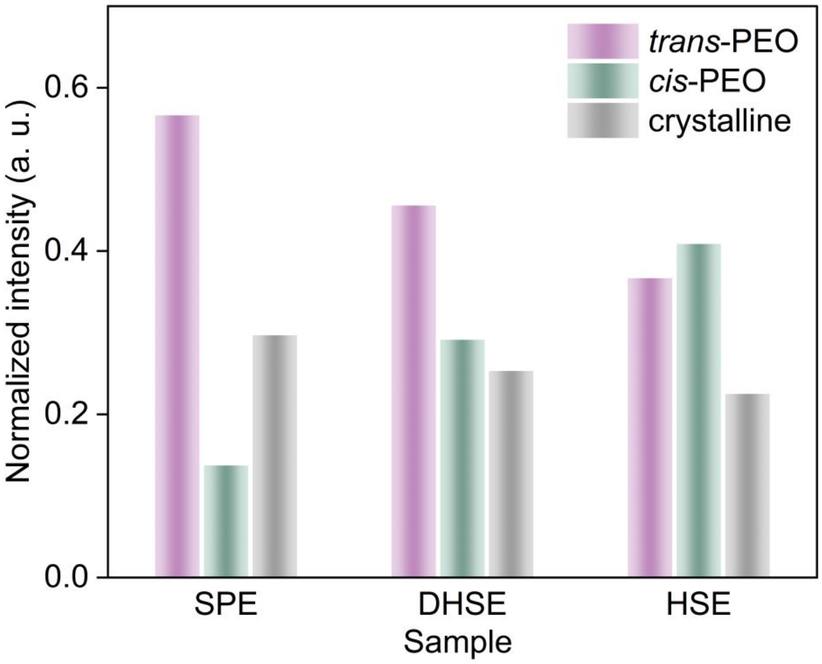

Figure S7. Characterizing the polymer conformation. Normalized peak intensity of the high power decoupling (hpdec) one-pulse ^{13}C MAS NMR spectra in **Figure 4a-c**. The data were fitted using MestReNova 11. The normalized intensity for each individual sample was derived from the peak areas obtained from the fits.

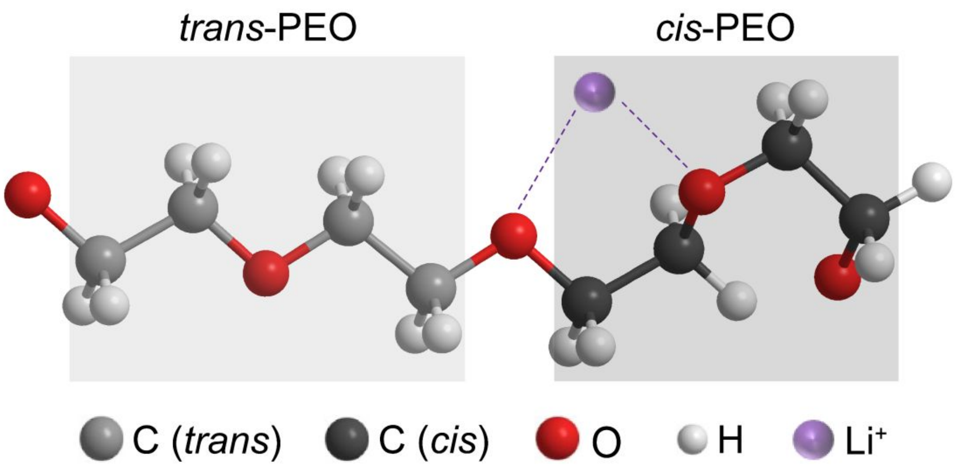

Figure S8. Carbon conformation diagram. Schematic showing the typical carbon conformation of the PEO chains.

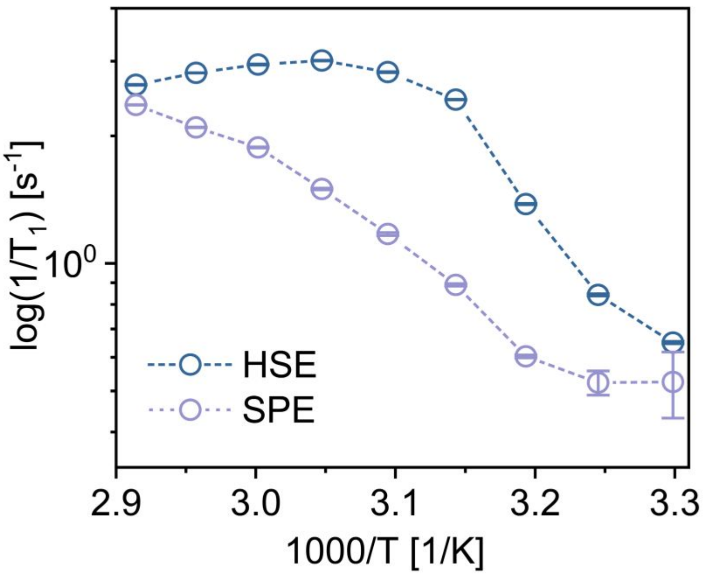

Figure S9. Probing local proton dynamics using ^{1}H T_1 relaxometry measurements. ^{1}H relaxation rates measured for the SPE and HSE.

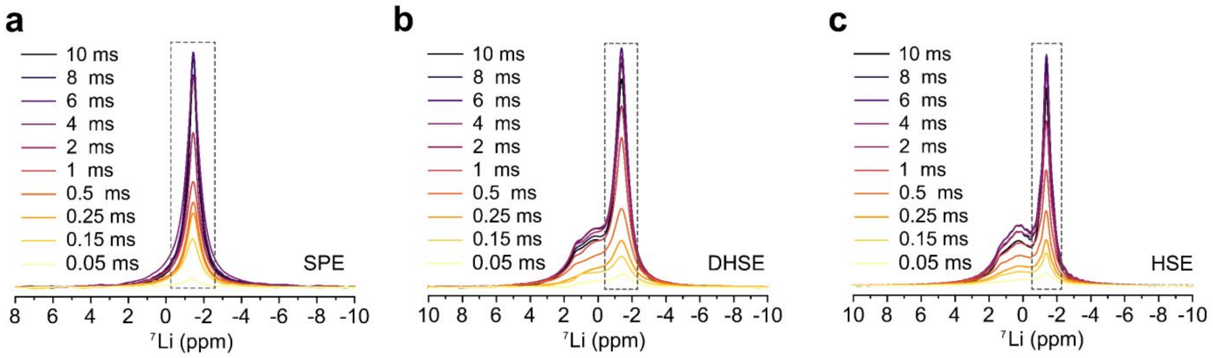

Figure S10. 1D ^{7}Li CP MAS spectra of the SPE and (D) HSE. 1D ^{7}Li CP MAS spectra of (**a**) SPE, (**b**) DHSE, and (**c**) HSE with contact times ranging from 0.05 to 10 ms. The LiTFSI-PEO peak is highlighted in the dotted box.

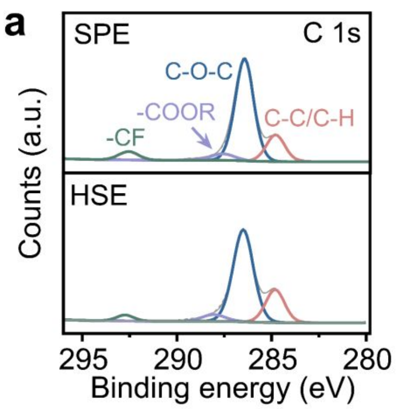

Figure S11. Characterizing the surface properties of the pristine SPE and HSE. XPS measurements of (**a**) C 1s, (**b**) F 1s, and (**c**) O 1s for the pristine SPE and HSE.

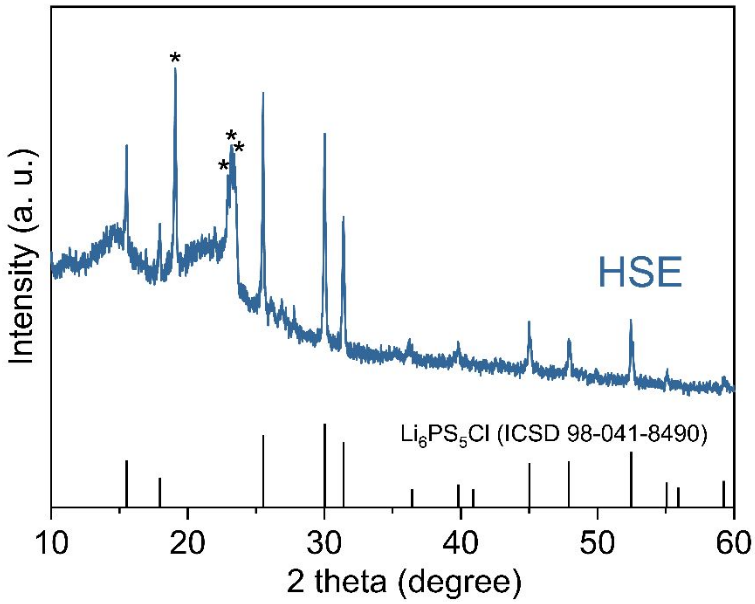

Figure S12. XRD pattern of the HSE. The XRD pattern of the HSE exhibits diffraction peaks that align well with the characteristic peaks of the crystalline Li_6PS_5Cl, with no noticeable peak shifts and broadening (additional peaks correspond to the PEO phase[13], indexed by asterisks), indicating the retention of the Li_6PS_5Cl crystalline phase.

Supplementary Tables

Table S1. Activation energies (E_a) obtained from the Hendrickson Bray and Abragam model fits of the ^{7}Li line-width measurements in Figure 2.

Sample	E_a from HB model (eV)	E_a from Abragam model (eV)	
		Arrhenuis fit	VTF fit
SPE	0.60 ± 0.03	0.89 ± 0.03	(13.3 ± 0.5) × 10^{-3}
HSE	0.51 ± 0.02	0.37 ± 0.01	(2.7 ± 0.1) × 10^{-3}
DHSE	0.54 ± 0.03	0.46 ± 0.01	(8.2 ± 0.5) × 10^{-3}

Table S2. Fitting results of the 1D ^{7}Li CP MAS curves in Figure S10.

Sample		T_{H-Li} (ms)	$T_{1\rho H}$ (ms)
SPE	LiTFSI-PEO	3.43 ± 0.18	83.54 ± 0.54
HSE	LiTFSI-PEO	1.15 ± 0.13	11.42 ± 0.65
DHSE	LiTFSI-PEO	1.59 ± 0.19	52.69 ± 0.42

Table S3. Fitting parameters obtained from the Hendrickson Bray's fits in Figure 2a,b (using Equation 5).

Sample	A (Hz)	B (Hz)	D (Hz)
SPE	5706 ± 59	(27.38 ± 1.13) × 10^{-7}	100 ± 32
HSE	5387 ± 79	(3.99 ± 0.07) × 10^{-7}	66 ± 29
DHSE	6262 ± 119	(1.18 ± 0.94) × 10^{-7}	120 ± 40

Reference

(1) Hendrickson, J. R.; Bray, P. J. A Phenomenological Equation for NMR Motional Narrowing in Solids. *J. Magn. Reson. (1969)* **1973**, *9* (3), 341–357.

(2) Abragam, A. *The Principles of Nuclear Magnetism*. The international series of monographs on physics. Clarendon Press: Oxford, 1989.

(3) Ries, M. E.; Klein, P. G.; Brereton, M. G.; Ward, I. M. Proton NMR Study of Rouse Dynamics and Ideal Glass Transition Temperature of Poly(Ethylene Oxide) LiCF3SO3 Complexes. *Macromolecules* **1998**, *31* (15), 4950–4956.

(4) Carvalho, L. M.; Guégan, P.; Cheradame, H.; Gomes, A. S. Variation of the Mesh Size of PEO-Based Networks Filled with TFSILi: From an Arrhenius to WLF Type Conductivity Behavior. *Eur. Polym. J.* **2000**, *36* (2), 401–409.

(5) Aziz, S. B.; Woo, T. J.; Kadir, M. F. Z.; Ahmed, H. M. A Conceptual Review on Polymer Electrolytes and Ion Transport Models. *J. Sci.: Adv. Mater. Devices* **2018**, *3* (1), 1–17.

(6) Levitt, M. H. *Spin Dynamics: Basics of Nuclear Magnetic Resonance*, 2nd ed.; John Wiley & Sons: Chichester, England, 2008.

(7) Beckmann, P. A. Spectral Densities and Nuclear Spin Relaxation in Solids. *Physics Reports* **1988**, *171* (3), 85–128.

(8) Hanghofer, I.; Brinek, M.; Eisbacher, S. L.; Bitschnau, B.; Volck, M.; Hennige, V.; Hanzu, I.; Rettenwander, D.; Wilkening, H. M. R. Substitutional Disorder: Structure and Ion Dynamics of the Argyrodites Li_6PS_5Cl, Li_6PS_5Br and Li_6PS_5I. *Phys. Chem. Chem. Phys.* **2019**, *21* (16), 8489–8507.

(9) Salamon, M. B., Ed. *Physics of Superionic Conductors*. Topics in Current Physics; Springer Berlin Heidelberg: Berlin, Heidelberg, 1979; Vol. 15.

(10) Liu, M., Zhang, S., van Eck, E. R., Wang, C., Ganapathy, S., & Wagemaker, M. Improving Li-Ion Interfacial Transport in Hybrid Solid Electrolytes. *Nat. Nanotechnol.* **2022**, *17* (9), 959–967.

(11) Babij, N. R.; McCusker, E. O.; Whiteker, G. T.; Canturk, B.; Choy, N.; Creemer, L. C.; Amicis, C. V. D.; Hewlett, N. M.; Johnson, P. L.; Knobelsdorf, J. A. NMR Chemical Shifts of Trace Impurities: Industrially Preferred Solvents Used in Process and Green Chemistry. *Org. Process Res. Dev.* **2016**, *20* (3), 661–667.

(12) Keeler, J. J. *Understanding NMR Spectroscopy*, 2nd ed., repr.; Wiley: Chichester, West Sussex, 2011.

(13) Marzantowicz, M.; Dygas, J. R.; Krok, F.; Nowiński, J. L.; Tomaszewska, A.; Florjańczyk, Z.; Zygadło-Monikowska, E. Crystalline Phases, Morphology and Conductivity of PEO:LiTFSI Electrolytes in the Eutectic Region. *J. Power Sources* **2006**, *159* (1), 420–430.
